# Integrated transcriptome and metabolome analyses provide molecular insights into the transition of flower color in the rose cultivar ‘Juicy Terrazza’

**DOI:** 10.1186/s12870-025-06794-2

**Published:** 2025-07-04

**Authors:** Yun Xuan, Jie Ren, Zhu Chen, Dan Shi

**Affiliations:** Institute of Agricultural Machinery Equipment and Engineering, Anhui Academy of Agricultural Sciences, Hefei, 230031 China

**Keywords:** Rose, Petal color, Transcriptome, Metabolome, Carotenoids, Flavonoids, Molecular regulation

## Abstract

**Background:**

Flower color is a prominent ornamental characteristic in roses, and their petals contain a wide range of bioactive compounds. In this study, the petal development of the rose cultivar ‘Juicy Terrazza’ was categorized into three stages: tangerine-colored petals in the flower bud (JT-T), orange-colored petals of the blooming flower (JT-O), and pink-colored petals of the open flower (JT-P). We utilized transcriptomics and metabolomics approaches to investigate the genes and metabolites involved in the pigment metabolic pathway across all three stages of rose petals (JT-T, JT-O, and JT-P) for transcriptomics analysis and two stages (JT-T and JT-P) for metabolomics analysis.

**Result:**

A total of 872 metabolites were identified in rose flowers. Comparative analysis revealed significant differences in 153 accumulated metabolites between open flowers in JT-P and flower buds in JT-T. The flower coloration of this rose cultivar is primarily influenced by carotenoids and anthocyanins, with carotenoids being the main differential metabolites responsible for altering the flower colors during the transition from JT-T to JT-P, particularly β-carotene, violaxanthin and its derivatives. Furthermore, by conducting a comparative study on differentially expressed genes (DEGs) during the transition of petal color, potential candidate genes related to this specific phenotypic characteristic were successfully identified. *PSY*, *PDS*, *ZISO*, and *ZDS* genes showed significant down-regulation, while *RcCCD4* exhibited strong up-regulation in JT-P compared with JT-T, which could directly contribute to the reduction of carotenoid contents during the JT-P stage. The TF-gene-metabolite correlations involved in the change of pigments in rose petals were identified through comprehensive data analysis. *MYB308* and *MYB1* (*RchiOBHmChr3g0448721*, *RchiOBHmChr2g0116041*) could play pivotal roles in the regulation of pigment metabolism in this rose cultivar.

**Conclusions:**

These findings contribute to our understanding of the impact of specific metabolites and transcripts on flower color changes and the molecular mechanisms of carotenoid metabolism and flavonoid biosynthesis in rose flowers. The candidate key genes related to pigment metabolism may serve as valuable genetic resources for molecular breeding of ornamental plants with specific flower colors.

**Supplementary Information:**

The online version contains supplementary material available at 10.1186/s12870-025-06794-2.

## Background

Plants are abundant in primary metabolites and secondary metabolites, including carbohydrates, amino acids, nucleotides, fatty acids, flavonoids, terpenoids, alkaloids, tannins and various other compounds. Flavonoids and carotenoids exhibit widespread distribution in plants. Flavonoids, a subclass of phenylpropanoids, exhibit a wide array of colors ranging from pale yellow, red, violet to blue [1]. Particularly, anthocyanins, a subclass of flavonoids, display a broad array of hues spanning from orange/red to violet/blue. Water-soluble anthocyanins are synthesized in the cytosol and localized within plant cell vacuoles, whereas yellow-to-red, lipid-soluble carotenoids, which belong to a subclass of terpenoids known as tetraterpenes, are synthesized and stored in plastids, and serve as essential components for photosynthesis in plant cells [1–4]. The pigments, including anthocyanins and carotenoids, are present in various floral organs, fruits, foliage, and seeds, among others [5–8].

The rose, a widely cultivated ornamental plant worldwide, encompasses over 35,000 cultivars to date [9]. Flower color is a pivotal characteristic of ornamental roses. Anthocyanins and carotenoids are primarily responsible for the diverse range of flower colors in roses. The red hue predominantly arises from anthocyanins, while the yellow hue mainly stems from carotenoids. The orange color results from the combined presence of both pigments [10]. The rose not only possesses high ornamental value, but also exhibits edibility, medicinal properties, and cosmetic applications [11–13]. It finds extensive usage in horticulture, food, pharmaceuticals, and cosmetics. Rose flowers have garnered significant attention as abundant sources of bioactive compounds [14]. They are particularly rich in various flavonoids such as flavonols, anthocyanins, flavones, etc., which confer pharmacological actions including antioxidant, anti-inflammatory, antiallergic and anticancer effects [13, 15–19]. The petals of yellow and orange roses contain abundant carotenoids, which also contribute to human health. However, there have been only a limited number of published reports on carotenoids in rose petals [5, 10, 14].

Regarding anthocyanin biosynthesis, the regulatory factors *MYB*, *bHLH*, and *WDR* play crucial roles in regulating flavonoid biosynthesis [20, 21] and also contribute to the regulation of carotenoid accumulation [22, 23]. Additionally, other transcription factors (*TFs*), such as *WRKY* and *AP2/ERF*, are also involved in flavonoid metabolism [20, 24]. The control of plant carotenoid metabolism can involve *TFs* such as *MYB*, *bHLH*, *bZIP*, *MADS*, *WRKY*, and *AP2/ERF* [23]. The carotenoid accumulation in flowers is regulated not only by structural genes related to carotenoid biosynthesis but also by various families of *TFs* including *R2R3-MYB*, *bHLH*, *AP2/EREBP*, and *WRKY* [25, 26]. A previous study has revealed that the transcription factor *RcMYB1* not only plays a pivotal role in regulating rose anthocyanin biosynthesis but also participates in the regulation of carotenoid metabolism [27].

Currently, the in-depth exploration of carotenoid compositions in rose petals, as well as transcription factors and structural genes regulating carotenoid metabolism in roses, remains limited. To further investigate the coloration mechanism of orange flowers in roses and clarify the correlation between anthocyanin and carotenoid accumulations and petal color changes, it is necessary to conduct a more extensive study on the metabolic and transcriptomic profiles of rose petals. The rose cultivar ‘Juicy Terrazza’, which originates from the Netherlands, exhibits tangerine (reddish orange), orange, and pink hues at different developmental stages. In this study, we analyzed both the metabolic and transcriptomic data obtained from flower buds and fully open flowers of this rose cultivar. We conducted a comparison of the expression levels of genes associated with carotenoid metabolism as well as those related to anthocyanin biosynthesis while assessing the accumulation contents of carotenoids and flavonoids at various stages. Furthermore, we investigated gene-to-metabolite linkages related to carotenoids and flavonoids in roses. This study aims to elucidate the changes in petal pigments along with the molecular regulatory mechanisms underlying flower color variation in roses.

## Materials and methods

### Plant materials

The rose cultivar ‘Juicy Terrazza’ (JT) plants were cultivated in experimental plots of the rose research base at the Anhui Academy of Agricultural Sciences, Hefei, China (E 117°25′, N 31°58′). Rose petals were harvested from plants in November 2020. Based on the noticeable alterations in petal color during rose flower blooming, the process was categorized into three stages: tangerine-colored petals in the flower bud (JT-T), orange-colored petals of the blooming flower (JT-O), and pink-colored petals of the open flower (JT-P). Petal samples gathered from three plants were considered as one mixed sample. These samples were collected using three independent biological replicates, and rapidly frozen in liquid nitrogen. All rose petal samples were stored at a temperature of -80℃ until further analysis.

### RNA extraction, cDNA library construction and RNA-seq

The RNA from rose petals was isolated using the Trizol reagent (Invitrogen, CA, USA). The quality of the extracted RNA was evaluated using an Agilent 2100 Bioanalyzer (Agilent Technologies, CA, USA) and visualized on a 1% agarose gel. Following total RNA extraction, rose mRNA was enriched utilizing Oligo(dT) beads. Subsequently, the enriched rose mRNA was fragmented into shorter fragments using a fragmentation buffer and reverse transcribed with random primers to generate cDNA. Purification of the cDNA library fragments was performed using the QiaQuick PCR Purification Kit (Qiagen, Venlo, The Netherlands), followed by end repair, addition of an A base, and ligation to Illumina sequencing adapters. The ligated products were then separated via agarose gel electrophoresis and subjected to PCR amplification prior to sequencing on an Illumina NovaSeq6000 platform.

### RNA-seq analysis

To ensure the production of clean reads for sequence assembly and analysis, an additional filtration step was performed on the raw reads utilizing fastp [28]. The filtration process consisted of three steps: removing all reads containing adapters, eliminating reads with more than 10% unknown nucleotides (N), and discarding low-quality reads with over 50% of bases having a Q-value ≤ 20. Then, the reference genome was used to align the paired-end clean reads with HISAT2.2.4 software [29]. Mapping and assembling of the mapped reads from each sample were conducted using StringTie v1.3.1 [30, 31]. RSEM software was used to calculate Fragments Per Kilobase of Transcript per Million Mapped Reads (FPKM) values for quantifying gene expression levels [32]. FPKM values of ≥ 1 in at least one sample were defined as the threshold for gene expression. Genes with a fold change (FC) of at least 2 and a false discovery rate (FDR) lower than 0.05 were identified as differentially expressed genes (DEGs). For reference transcriptome sequencing, *Rosa chinensis* ‘Old Blush’ was employed as the source for the reference genome [33]. Subsequently, gene annotation was performed, followed by the analysis of Gene Ontology (GO) enrichment and KEGG pathway enrichment.

### Extraction and relative quantitative analysis of metabolites

The freeze-dried samples were pulverized using a zirconia bead mill (30 Hz, 1.5 min). Subsequently, the resulting powder was weighed, and 100 mg of it underwent an overnight extraction at a temperature of 4 °C using 1.0 ml of 70% aqueous methanol. Following centrifugation at 10,000 ×g for 10 min, the supernatant was filtered through a SCAA-104 membrane with a 0.22 μm pore size. The compounds extracted were analyzed using an LC-ESI-MS/MS system [34], and the obtained metabolites were identified by internal and public metabolite databases (MassBank, KNAPSAcK, HMDB, MoToDB, and METLIN). After acquiring the metabolic MS data for each sample, peak area integration was conducted to determine the relative content of each metabolite based on its corresponding chromatographic peaks.

Based on the comprehensive metabolite profile of the rose petals, principal component analysis (PCA) was employed for unsupervised dimensionality reduction using R package models. Orthogonal projections to latent structures discriminant analysis (OPLS-DA) was then used to assess the differences between distinct groups.

The criteria of T-test *P* < 0.05 and VIP ≥ 1 were utilized to identify differentially accumulated metabolites (DAMs) in this study.

### Extraction and quantitative analysis of carotenoid metabolites

The rose petal powder (50 mg) was extracted using a solution composed of n-hexane, acetone, and ethanol in equal proportions (1:1:1, v/v/v), supplemented with 10 µL of an internal standard mixed solution. The extraction process involved vortexing the mixture for 20 min at room temperature. After centrifugation at 12,000 r/min for 5 min at 4 °C, the supernatants were collected. The residue was then re-extracted following the same steps and conditions. These combined supernatants were evaporated to dryness and then reconstituted in a solution containing equal parts of MeOH and MTBE (1:1, v/v). Finally, the solution underwent filtration using a 0.22 μm membrane filter in preparation for subsequent LC-MS/MS analysis [35–37]. The sample extracts were analyzed using a UPLC-APCI-MS/MS system [36, 38]. Multiquant 3.0.3 software (Sciex) was utilized for metabolite quantification.

The R package pheatmap was utilized to generate heatmaps with dendrograms, visually representing the hierarchical cluster analysis (HCA) outcomes for both samples and metabolites. Normalized signal intensities of metabolites (unit variance scaling) were represented by a color spectrum. Significantly regulated carotenoids and derivatives between groups were identified based on an absolute Log_2_FC (fold change) ≥ 1 and a T-test P-value < 0.05.

### Integrative analysis of transcriptomic and metabolomic data

The Pearson correlation coefficients were computed to perform a comprehensive analysis of the transcriptome and metabolome data. Correlations with a coefficient|r| greater than 0.8 and a P-value less than 0.05 were deemed statistically significant. Transcriptomic and metabolomic data from both the JT-T and JT-P stages were integrated for gene-metabolite network analysis. Finally, the relationships between the transcriptome and metabolome were visualized using Cytoscape software (version 3.10.1).

## Results

### Summary of transcriptome sequencing of the rose flowers

**Fig. 1:**
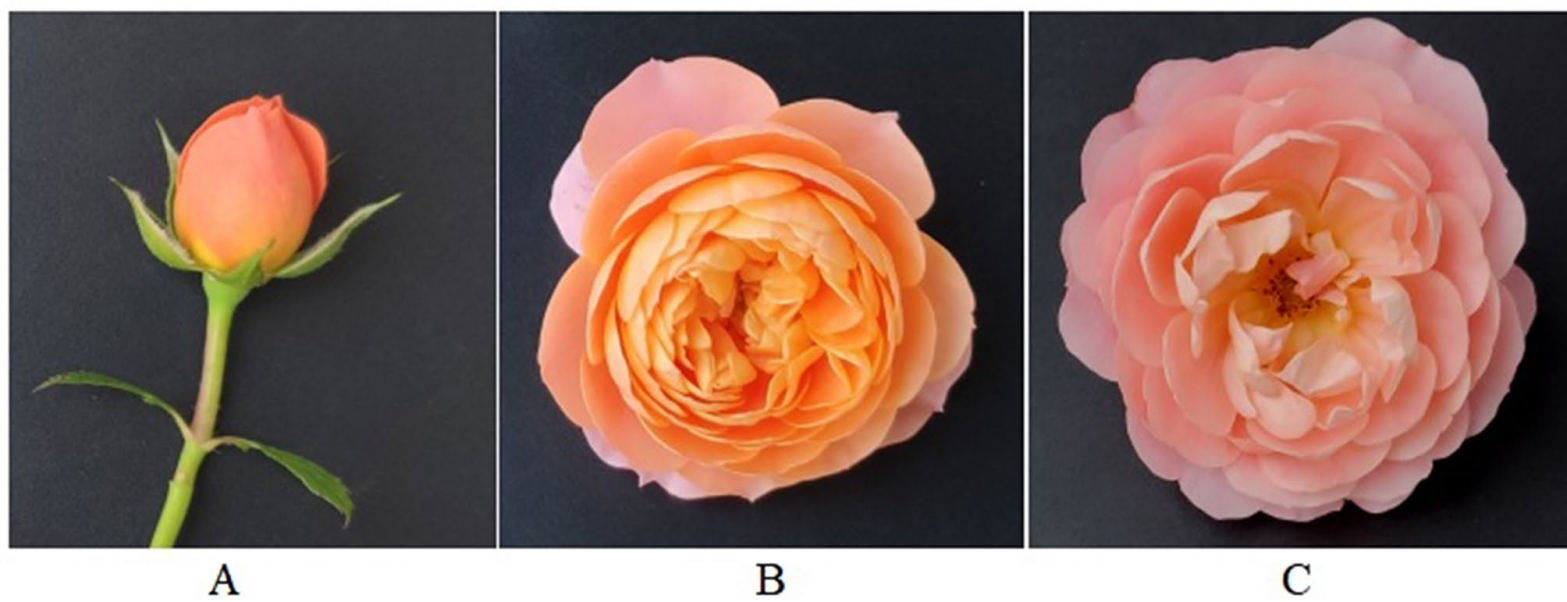
Three stages of petal development in the rose cultivar ‘Juicy Terrazza’. **A**. JT-T, tangerine petals in the flower bud. **B**. JT-O, orange petals of the blooming flower. **C**. JT-P, pink petals of the open flower

The petals of ‘Juicy Terrazza’ flowers were categorized into three distinct developmental stages: tangerine petals in the bud (JT-T), orange petals of the blooming flower (JT-O), and pink petals of the open flower (JT-P) (Fig. 1). The JT-T petals exhibited a small size, high pigment accumulation, and tight arrangement. Subsequently, as the petals expanded, there was a noticeable change in pigmentation, resulting in an orange blossom. Approximately four days later, the open flower (JT-P) displayed pink-colored petals.

The RNA extracted from the petals of ‘Juicy Terrazza’ flowers at three different developmental stages was subjected to sequencing using the Illumina NovaSeq 6000 platform. A total of 503,119,412 paired-end high-quality clean reads were obtained through RNA-seq analysis, with 177,345,636 reads for the JT-T sample, 169,134,444 reads for the JT-O sample, and 156,639,332 reads for the JT-P sample (Table S1). A total of approximately 74.99 Gb of clean data were generated, averaging approximately 8.33 Gb per sample. The Q30 value was determined to be 92.39%, while the average GC content was found to be approximately 45.58% (Table S1). Notably, more than 85% of these reads could be successfully mapped to reference sequences derived from *R. chinensis* in all nine samples (Table S2).

### Identification of differentially expressed genes (DEGs) during the transition of petal color

**Fig. 2:**
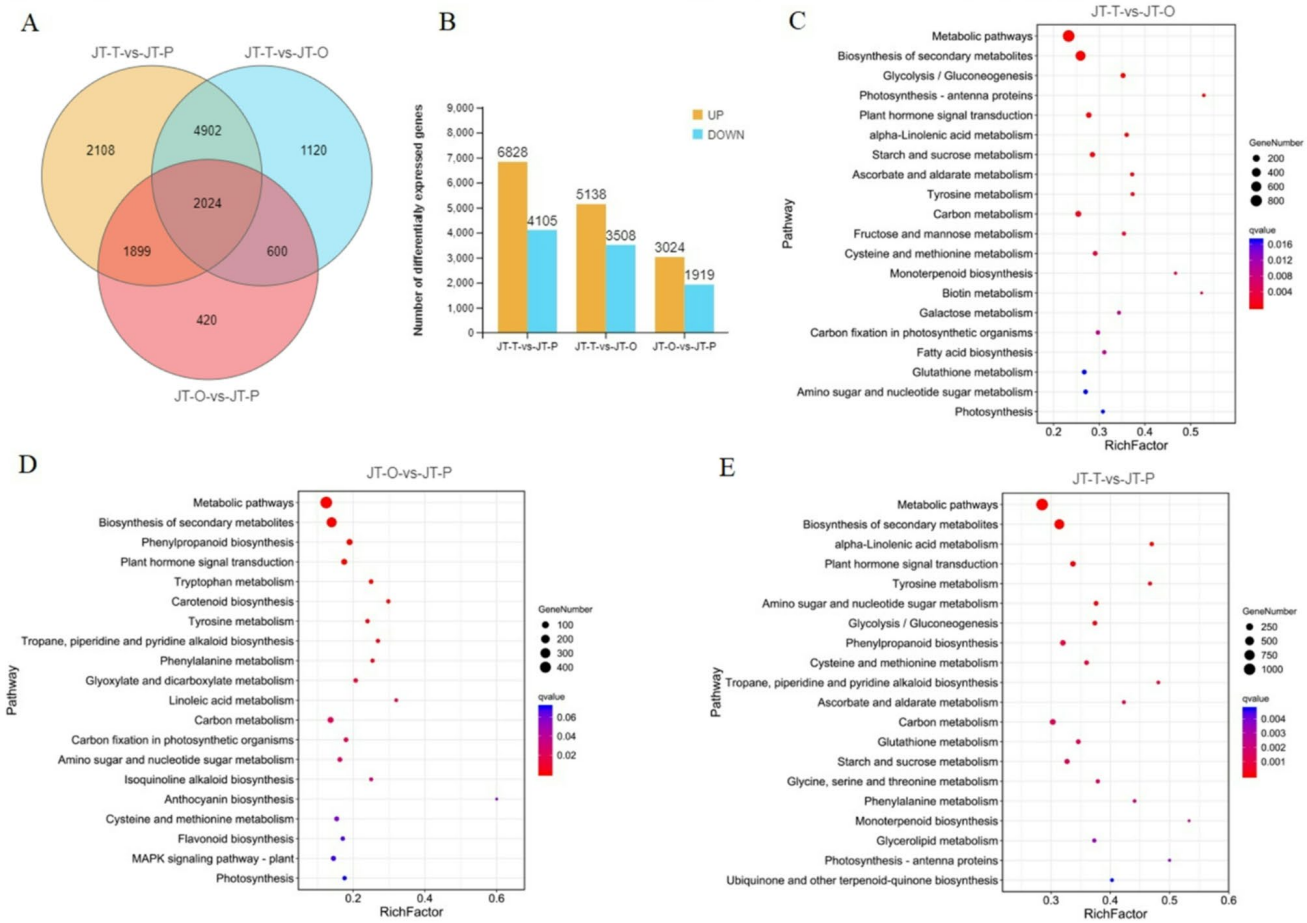
Comparative analysis of DEGs from rose petals at the three developmental stages. **A**.Venn diagram showing the number of DEGs among the three comparisons: JT-T vs. JT-P, JT-T vs. JT-O and JT-O vs. JT-P. **B**. The number of DEGs among the three comparisons. **C**. KO category enrichment of DEGs in the JT-T vs. JT-O comparison. **D**. KO category enrichment of DEGs in the JT-O vs. JT-P comparison. **E**. KO category enrichment of DEGs in the JT-T vs. JT-P comparison

A thorough comparative analysis was performed to investigate the DEGs across three stage combinations (JT-T vs. JT-O, JT-O vs. JT-P, and JT-T vs. JT-P), which indicated notable alterations in petal colors. JT-T vs. JT-O denotes the process during which tangerine petal growth primarily occurs through cell expansion until the flower blossoms. JT-O vs. JT-P denotes the process during which petal colors undergo significant changes from orange to pink. Lastly, JT-T vs. JT-P denotes the process during which petal colors undergo a substantial transformation from tangerine to pink. There were 8,646 DEGs, 4,943 DEGs, and 10,933 DEGs specifically in these three comparisons, respectively (Fig. 2A). In the JT-T vs. JT-O comparison, 5,138 DEGs were up-regulated and 3,508 DEGs were down-regulated. In JT-O vs. JT-P, 3,024 DEGs were up-regulated and 1,919 DEGs were down-regulated. In the comparison between JT-T and JT-P, a total of 6,828 DEGs were up-regulated and 4,105 DEGs were down-regulated (Fig. 2B). These findings indicate that during floral opening of ‘Juicy Terrazza’, there is a relatively higher number of up-regulated DEGs compared to down-regulated ones.

The 24,522 DEGs obtained from the three comparisons were classified into three distinct Gene Ontology (GO) categories (biological process, molecular function, and cellular component) (Fig. S1). Functional analysis of these DEGs revealed enrichment in eighteen primary biological processes, such as metabolic process, cellular process, and single-organism process. Within the cellular component category, these DEGs showed enrichment in nine major groups such as cell, cell part, and organelle. In terms of the molecular function category, most of the DEGs displayed enrichment in catalytic activity, binding and transporter activity. KEGG analysis revealed that photosynthesis-antenna proteins and glycolysis/gluconeogenesis were the two primary enriched pathways in the JT-T vs. JT-O comparison (Fig. 2C). This finding suggests that as the petals gradually unfold, photosynthesis-antenna proteins may enhance their roles in light signal perception or photoprotection, while the glycolysis and sugar metabolic synthesis pathways are enriched to support the expansion of petal cells. Meanwhile, phenylpropanoid biosynthesis, plant hormone signal transduction, tryptophan metabolism and carotenoid biosynthesis were the main enriched pathways in the JT-O vs. JT-P comparison (Fig. 2D), suggesting that these enriched pathways potentially play crucial roles during the transformation process of petal pigments and physiological conditions. Additionally, alpha-linolenic acid metabolism was the most enriched pathway in the JT-T vs. JT-P comparison (Fig. 2E), suggesting that this metabolic pathway may be closely linked to the regulation of petal development, and potentially participate in aging control and defense responses.

### Transcriptomic analysis of transcription factors (TFs) during the transition of petal color

**Fig. 3:**
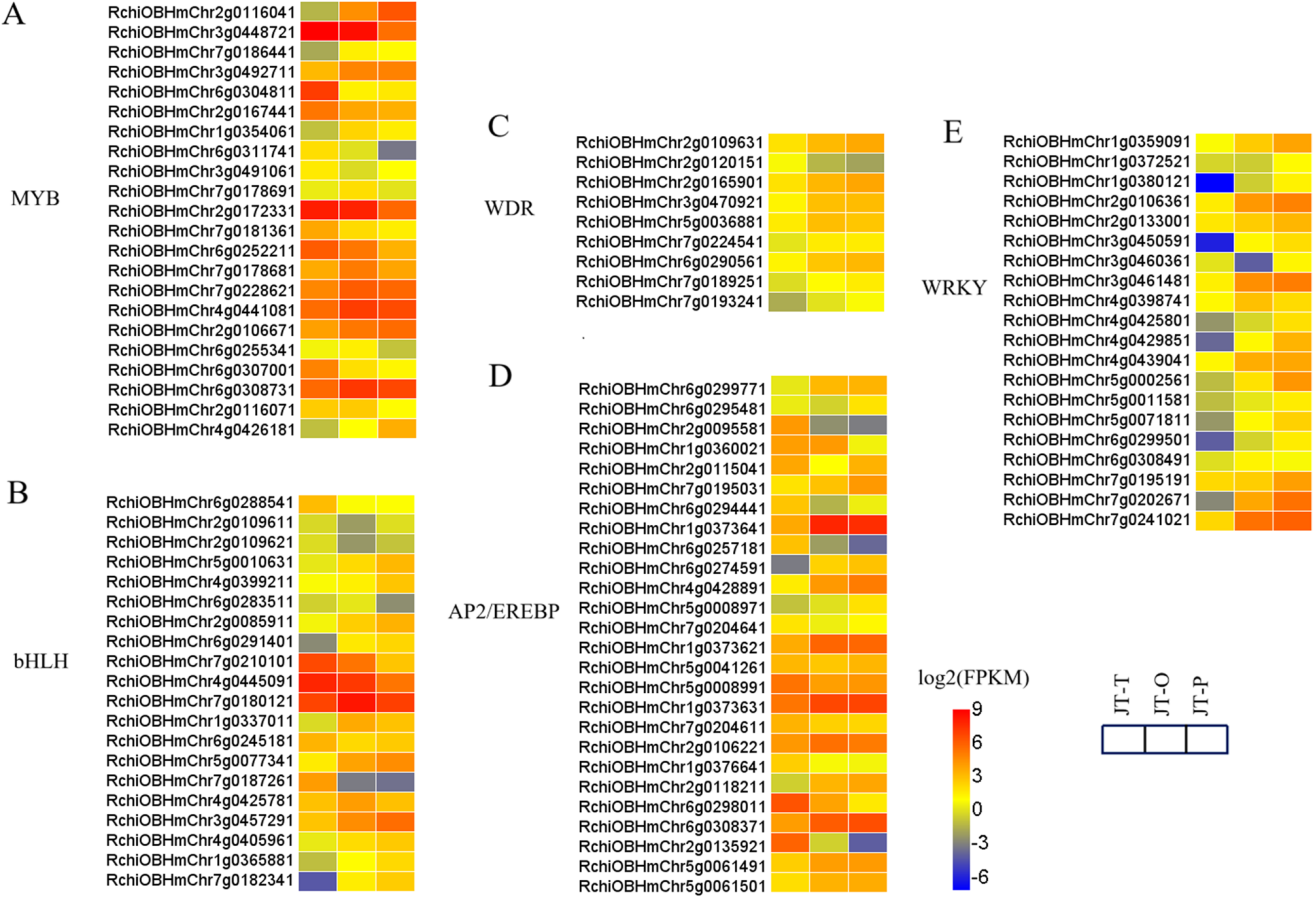
Heatmaps illustrating DEGs based on log2(FPKM) values for five different families of transcription factors (TFs) during the petal color transition phase. (**A**) MYB transcription factors. (**B**) bHLH transcription factors. (**C**) WDR transcription factors. (**D**) AP2/EREBP transcription factors. (**E**) WRKY transcription factors. The color scale indicates log2(FPKM) values

Previous studies have demonstrated that transcription factors including *MYB*, *bHLH*, *AP2/EREBP*, *WDR*, and *WRKY* play a crucial role in regulating the accumulation of flavonoids and carotenoids in flowers [20–27]. In this study, these families of differentially expressed *TFs* in the transcriptome of rose flowers are listed in Table S3, with 97 genes observed during the petal color change in rose cultivar ‘Juicy Terrazza’, as shown in Fig. 3. These DEGs included 22 *MYBs*, 20 *bHLHs*, 9 *WDRs*, 26 *AP2/EREBPs* and 20 *WRKYs*. In JT-T vs. JT-O, the expression levels of 11 *MYBs*, 11 *bHLHs*, 8 *WDRs*, 12 *AP2/EREBPs* and 16 *WRKYs* were significantly up-regulated, while 6 *MYBs*, 6 *bHLHs*, 1 WDR, 9 *AP2/EREBPs*, and 1 *WRKY* were remarkably downregulated. Additionally, in JT-O vs. JT-P, 3 *MYBs*, 6 *bHLHs*, 5 *AP2/EREBPs*, and 12 *WRKYs* were significantly upregulated, while 8 *MYBs*, 5 *bHLHs*, and 4 *AP2/EREBPs* were remarkably down-regulated. In JT-T vs. JT-P, 9 *MYBs*, 10 *bHLHs*, 8 *WDRs*, 13 *AP2/EREBPs* and 18 *WRKYs* were up-regulated, while 10 *MYBs*, 5 *bHLHs*, 1 *WDR* and 9 *AP2/EREBPs* were remarkably down-regulated.

Moreover, the majority of transcription factors (*TFs*) exhibited significant up-regulation in JT-T vs. JT-P. The expression levels of *MYBs* (*RchiOBHmChr2g0116041* and *RchiOBHmChr4g0426181*) in rose petals exhibited a significant increase, exceeding twenty-fold in JT-P, compared to JT-T. Conversely, *RchiOBHmChr3g0448721*, *RchiOBHmChr6g0311741*, *RchiOBHmChr6g0304811*, and *RchiOBHmChr6g0307001* displayed a more than ten-fold decrease in expression. 5 *bHLHs* were up-regulated more than eight-fold while 2 *bHLHs* were down-regulated by the same magnitude. In contrast, 5 *AP2/EREBPs* were up-regulated more than eight-fold whereas 5 *AP2/EREBPs* were down-regulated similarly. Furthermore, 12 *WRKYs* exhibited more than eight-fold up-regulation with no significant down-regulation observed among *WRKYs*. In summary, these differentially expressed transcription factors are potential candidate genes involved in regulating anthocyanin, carotenoid metabolism and petal development in rose.

### Quantitative analysis of the metabolites of the rose petals using widely targeted metabolomics

**Fig. 4:**
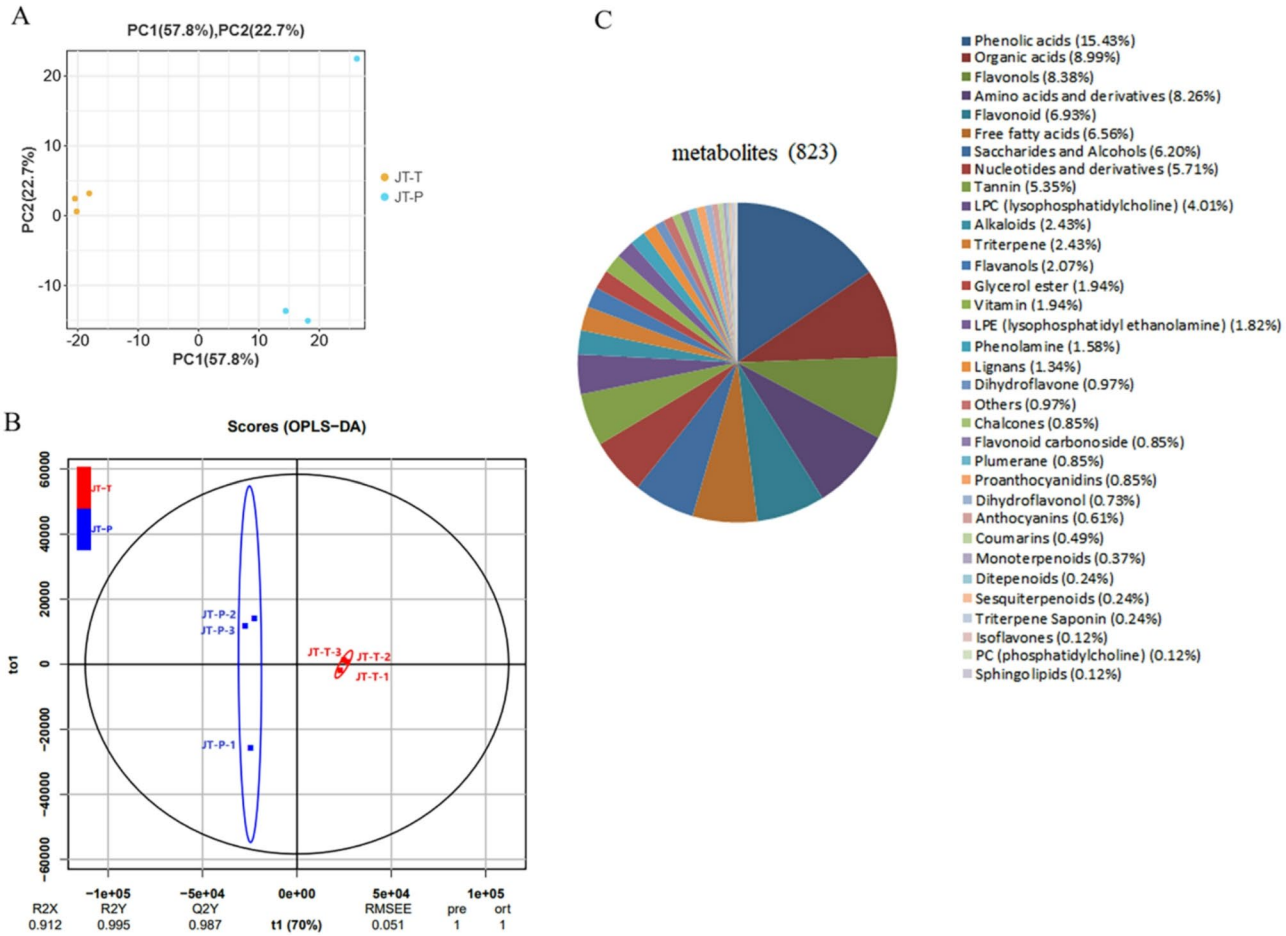
Comparative analysis of metabolites from rose petals at two stages (JT-T and JT-P). **A**. PCA score plots of JT-T vs. JT-P. **B**. OPLS-DA of JT-T vs. JT-P. **C**. Functional categories of the identified metabolites in rose petals

In our research, the rose buds in JT-T showed tangerine-colored petals, and the open flowers in JT-P displayed pink petals, while the blooming flowers in JT-O exhibited an overall orange color with pink outer petals. Therefore, we chose to compare the differences in metabolite composition between rose petals at the flower bud (JT-T) and open flower (JT-P) stages. Petal samples were subjected to UPLC-ESI-MS/MS analysis based on extensive targeted metabolomics. The PCA is used to reveal the metabolic differences between the groups and the variability among the intragroup samples. In Fig. 4A, the PCA score plot demonstrated the distinct separation between the JT-T and JT-P groups (PC1 = 57.8%, PC2 = 22.7%). The OPLS-DA score plot in Fig. 4B illustrates the discrimination between the JT-T and JT-P groups, with R^2^X = 0.912, R^2^Y = 0.995, and Q^2^ = 0.987. The high Q^2^ value (> 0.9) indicates that the model is reliable and stable for comparing metabolite accumulation between the JT-T and JT-P groups. Overall, these results highlight notable variations in the accumulation of metabolites between the two groups.

Additionally, the identified metabolites in rose petals at the JT-T and JT-P stages were visualized through a heatmap (Fig. S2). In total, the 823 identified metabolites were classified into 34 categories based on their functions (Fig. 4C). The detailed information can be found in Table S4. These metabolites included 127 phenolic acids, 74 organic acids, 69 flavonols, 68 amino acids and derivatives, 57 flavonoids, 54 free fatty acids, 51 alcohols and saccharides, 47 nucleotides and derivatives, 44 tannins, 33 LPCs (lysophosphatidylcholines), 20 alkaloids, 20 triterpenes, 17 flavanols, 16 vitamins, 8 dihydroflavones, 7 chalcones, 5 anthocyanins, among others.

**Fig. 5:**
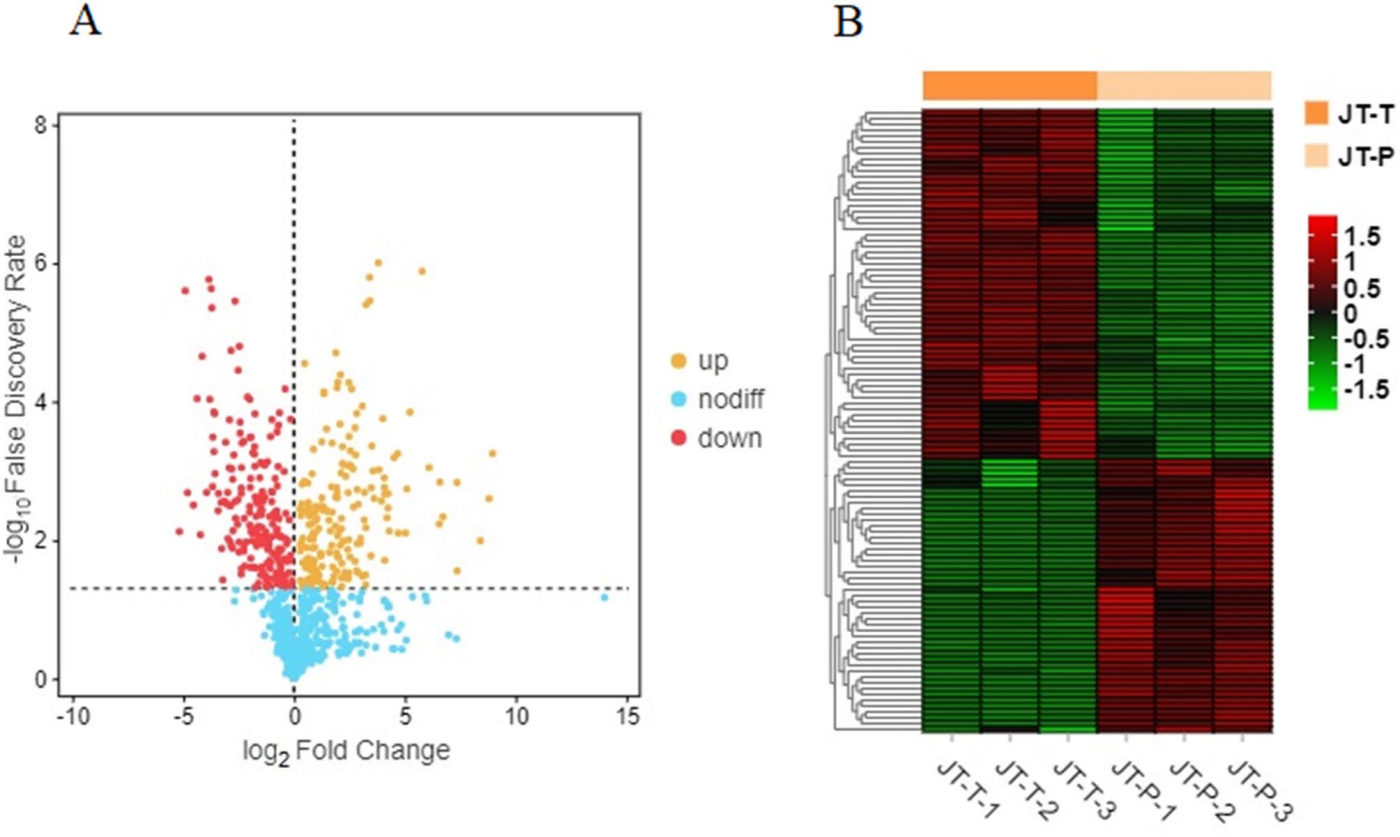
Comparative analysis of differentially accumulated metabolites (DAMs) from rose petals at two stages (JT-T and JT-P). **A**. Volcano plots of DAMs in JT-T vs. JT-P. Orange dots represent up-regulated DAMs, red dots indicate down-regulated DAMs, and blue dots denote non-differentially accumulated metabolites. **B**. A heatmap of 107 DAMs from rose petals at two stages (JT-T and JT-P). The color scale (from red to green) indicates the normalized metabolite contents using the row Z-score

The volcano plot depicted the overall distribution of 823 metabolites between the JT-T and JT-P groups, where orange dots represented up-regulated DAMs, red dots indicated down-regulated DAMs, and blue dots denoted nondifferentially accumulated metabolites (Fig. 5A). Additionally, a heatmap depicting DAMs in rose petals at two distinct stages was presented in Fig. 5B. A total of 107 DAMs were detected between the JT-T and JT-P groups, with 60 down-regulated metabolites and 47 up-regulated metabolites observed in the JT-P group compared to the JT-T group (Table 1 and Table S5).

The identified DAMs in the comparison of JT-T vs. JT-P could be classified into more than twelve classes, with a majority falling into seven categories including flavonoids (24.30%), tannins (14.02%), phenolic acids (12.15%), organic acids (11.21%), amino acids and derivatives (11.21%), alkaloids (8.41%), and nucleotides and derivatives (7.48%) respectively (Table 1). Among these, flavonoids further comprise seventeen flavonols, six flavones, one anthocyanin, one flavanol, and one dihydroflavonol (Table S5). The concentrations of most components belonging to the four classes of DAMs, such as flavonoids, tannins, organic acids, nucleotides and derivatives, were found to be higher in JT-T buds than in JT-P flowers.

Notably, dihydrokaempferol, gallic acid, guanosine, citric acid, and isocitric acid exhibited significantly decreased levels in JT-P flowers. Conversely, the concentrations of most components belonging to alkaloids and amino acid derivatives displayed markedly greater abundance in open flowers than in flower buds; for instance, piperidine, methylnicotinate, and glutathione (reduced form) showed notable increases. Furthermore, the content of L-ascorbic acid was exceptionally higher in JT-P flowers than in JT-T buds.

**Table 1:** Statistics of differentially accumulated metabolites in JT-T vs. JT-P

Class	Number (JT-T vs. JT-*P*)	
	up	down
Flavonoids	7	19
Tannins	1	14
Phenolic acids	7	6
Organic acids	4	8
Amino acids and derivatives	11	1
Alkaloids	6	3
Nucleotides and derivatives	2	6
Lignans and coumarins	2	0
Saccharides and Alcohols	2	1
Terpenoids	2	0
Vitamin	2	0
Lipids	1	1
Others	0	1
Total	47	60

### Quantitative analysis of the carotenoids in the rose petals

**Fig. 6:**
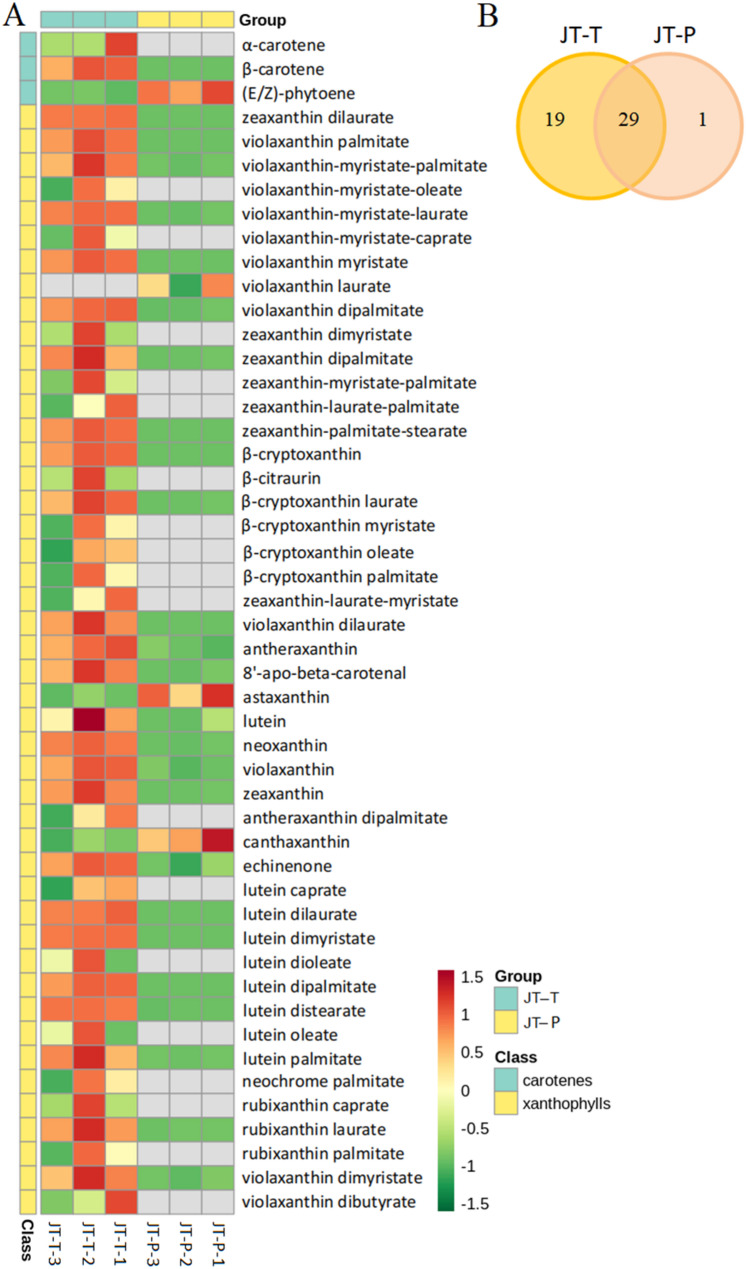
A preliminary analysis of carotenoids and their derivatives. **A**. A heat map of carotenoids and their derivatives in JT-T and JT-P. The color scale (from red to green) indicates the normalized metabolite contents using the row Z-score. **B**. Venn diagram illustrating the distribution of carotenoids and their derivatives in JT-T and JT-P

The carotenoids present in the rose petals of JT-T and JT-P stages were analyzed utilizing UPLC-MS/MS. In this research, a total of 49 carotenoids and their derivatives were identified, including 3 carotenes, 11 xanthophylls, and 35 xanthophyll derivatives. Specifically, 48 carotenoid-related metabolites were found in the petals of the JT-T stage while only 30 were identified in the petals of the JT-P stage (Fig. 6A). Additionally, there were 19 unique metabolites detected exclusively in the petals of the JT-T stage, only one specific metabolite found in the petals of the JT-P stage, and 29 metabolites detected in the petals of both the JT-T and JT-P stages (Fig. 6B).

The quantitative content analysis is presented in Table S6. Notably, the overall carotenoid content was significantly higher in the petals at the JT-T stage (317.62 µg/g FW) compared to those at the JT-P stage (51.18 µg/g FW). 25 carotenoids and derivatives were significantly lower (≥ 2 fold) in the petals at the JT-P stage. Furthermore, five carotenoids, namely α-carotene, canthaxanthin, echinenone, astaxanthin, and β-citraurin were identified in rose petals. Violaxanthin and its derivatives accounted for approximately 71.21% of total carotenoids, followed by β-carotene (9.43%), lutein and its derivatives (6.23%), β-cryptoxanthin (4.31%), and zeaxanthin with its derivatives (3.73%) as major components at the JT-T stage. Conversely, there was a lower presence of violaxanthin and its derivatives, along with lutein and β-carotene at the JT-P stage; α-cryptoxanthin was not detected in rose petals during these stages. Violaxanthin laurate was not detected in the petals of the JT-T stage; however, it was present in the petals of the JT-P stage. In comparison to the petals at the JT-T stage, there was a notable reduction in the content of various carotenoid compounds and total carotenoids in the petals at the JT-P stage, except for (E/Z)-phytoene, astaxanthin, lutein, and canthaxanthin. The concentrations of β-carotene, violaxanthin and its derivatives, as well as total carotenoids and their derivatives in the petals at the JT-T stage were 23-fold, 7.2-fold, and 6.2-fold higher, respectively, compared to those at the JT-P stage. Therefore, the degradation of carotenoids can be considered a primary factor contributing to the color change in rose petals.

### Carotenoid metabolic pathway

**Fig. 7:**
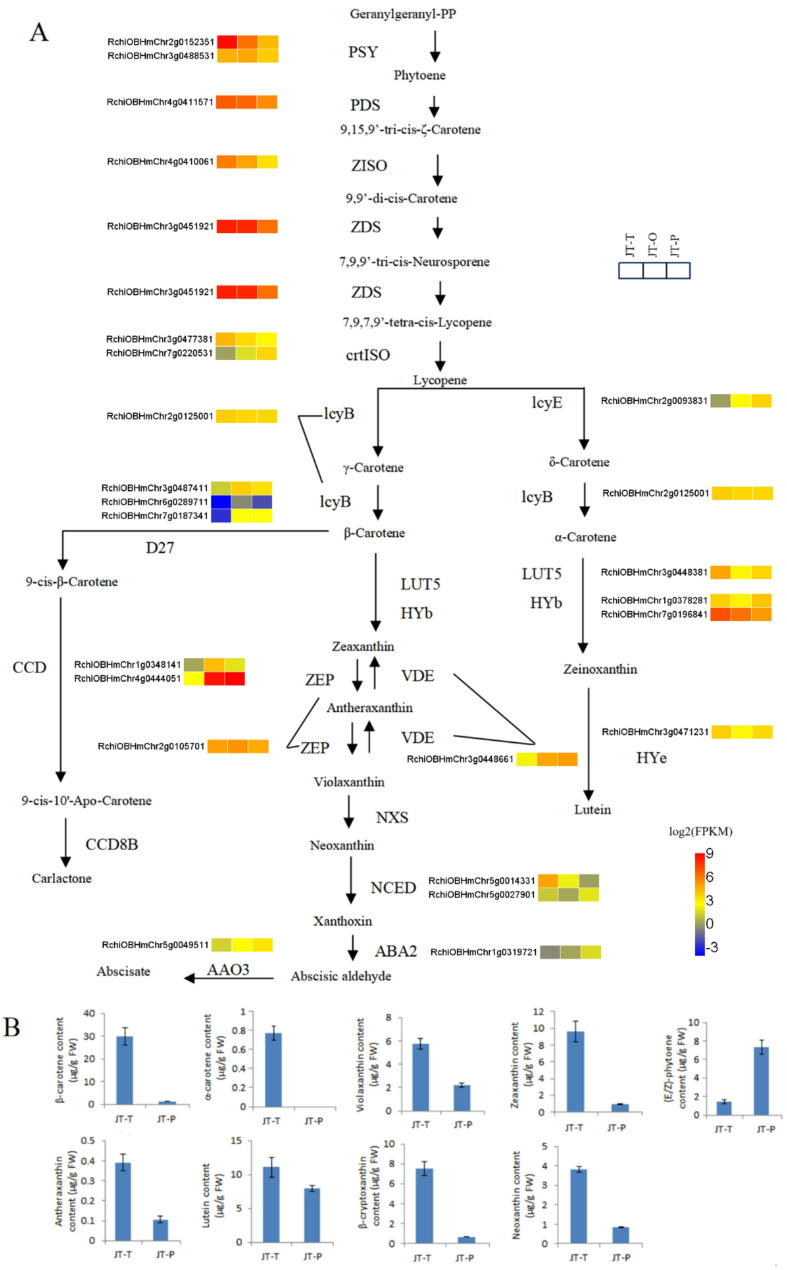
Metabolic pathway of carotenoids. **A**. Heatmaps illustrating the expression of genes related to the carotenoid metabolic pathway in rose petals. PSY, phytoene synthase; PDS, phytoene desaturase; ZISO, zeta-carotene isomerase; ZDS, zeta-carotene desaturase; crtISO, carotenoid isomerase; lcyB, lycopene beta cyclase; lcyE, lycopene epsilon cyclase; LUT5, beta-ring hydroxylase; HYb, beta-carotene hydroxylase; HYe, carotenoid epsilon hydroxylase; VDE, violaxanthin de-epoxidase; ZEP, zeaxanthin epoxidase; NXS, neoxanthin synthase; D27, beta-carotene isomerase; CCD, carotenoid cleavage dioxygenase; CCD8B, carlactone synthase; NCED, 9-cis-epoxycarotenoid dioxygenase; ABA2, xanthoxin dehydrogenase; AAO3, indole-3-acetaldehyde oxidase. The color scale indicates log2(FPKM) values. **B**. Carotenoids accumulation in JT-T and JT-P

Based on the metabolomic analysis results mentioned above, it was observed that the petals of rose cultivar ‘Juicy Terrazza’ contain key metabolites, such as carotenoids and flavonoids at the JT-T and JT-P stages, which directly influence flower color. Firstly, we conducted an analysis of carotenoid metabolism. In this pathway, there were 24 genes (21 DEGs) encoding 17 enzymes and eight DAMs associated with the carotenoid metabolic pathway (Fig. 7, Tables S6, S7). Specifically, in the lycopene formation step, most DEGs showed significant down-regulation in the comparisons of JT-O vs. JT-P and JT-T vs. JT-P stages. These DEGs included *PSY* (*RchiOBHmChr2g0152351*), *PDS* (*RchiOBHmChr4g0411571*), *ZISO* (*RchiOBHmChr4g0410061*), *ZDS* (*RchiOBHmChr3g0451921*) and *crtISO* (*RchiOBHmChr3g0477381*), except for *crtISO* (*RchiOBHmChr7g0220531*). Subsequently, lycopene was catalyzed by lycopene beta-cyclase and lycopene epsilon cyclase to produce β-carotene and α-carotene.

During the formation of 9-cis-β-Carotene, three D27 genes exhibited significant up-regulation in both comparisons: JT-T vs. JT-O and JT-T vs. JT-P. These genes are *RchiOBHmChr3g0487411*, *RchiOBHmChr6g0289711* and *RchiOBHmChr7g0187341*. Carotenoid cleavage dioxygenase (CCD) family including CCD4 and CCD7 catalyzes oxidative cleavage of carotenoids. The highly expressed probable *CCD4* gene (*RchiOBHmChr4g0444051*) was significantly up-regulated by more than 66-fold in the comparisons of JT-T vs. JT-O and JT-T vs. JT-P, while the *CCD7* gene (*RchiOBHmChr1g0348141*) exhibited a remarkable upregulation of over 15-fold in JT-T vs. JT-O. Violaxanthin and antheraxanthin were enzymatically de-epoxidized by violaxanthin de-epoxidase (VDE) to yield zeaxanthin. The *VDE* gene (*RchiOBHmChr3g0448661*) exhibited a significant upregulation of 5.8-fold and 7.1-fold in JT-O and JT-P, respectively, compared to JT-T.

In terms of metabolic aspect, compared with JT-T stage, most of the detected DAMs were greatly reduced in the petals of the JT-P stage except for phytoene. These metabolites included β-carotene, violaxanthin, β-cryptoxanthin, zeaxanthin, neoxanthin, and antheraxanthin. Furthermore, at the JT-T stage, petals exhibited a high concentration of β-carotene (29.96 µg/g FW) but contained relatively low levels of α-carotene (0.77 µg/g FW). In contrast, petals at the JT-P stage exhibited low levels of β-carotene (1.29 µg/g FW) and undetectable levels of α-carotene.

### Flavonoid biosynthetic pathway

**Fig. 8:**
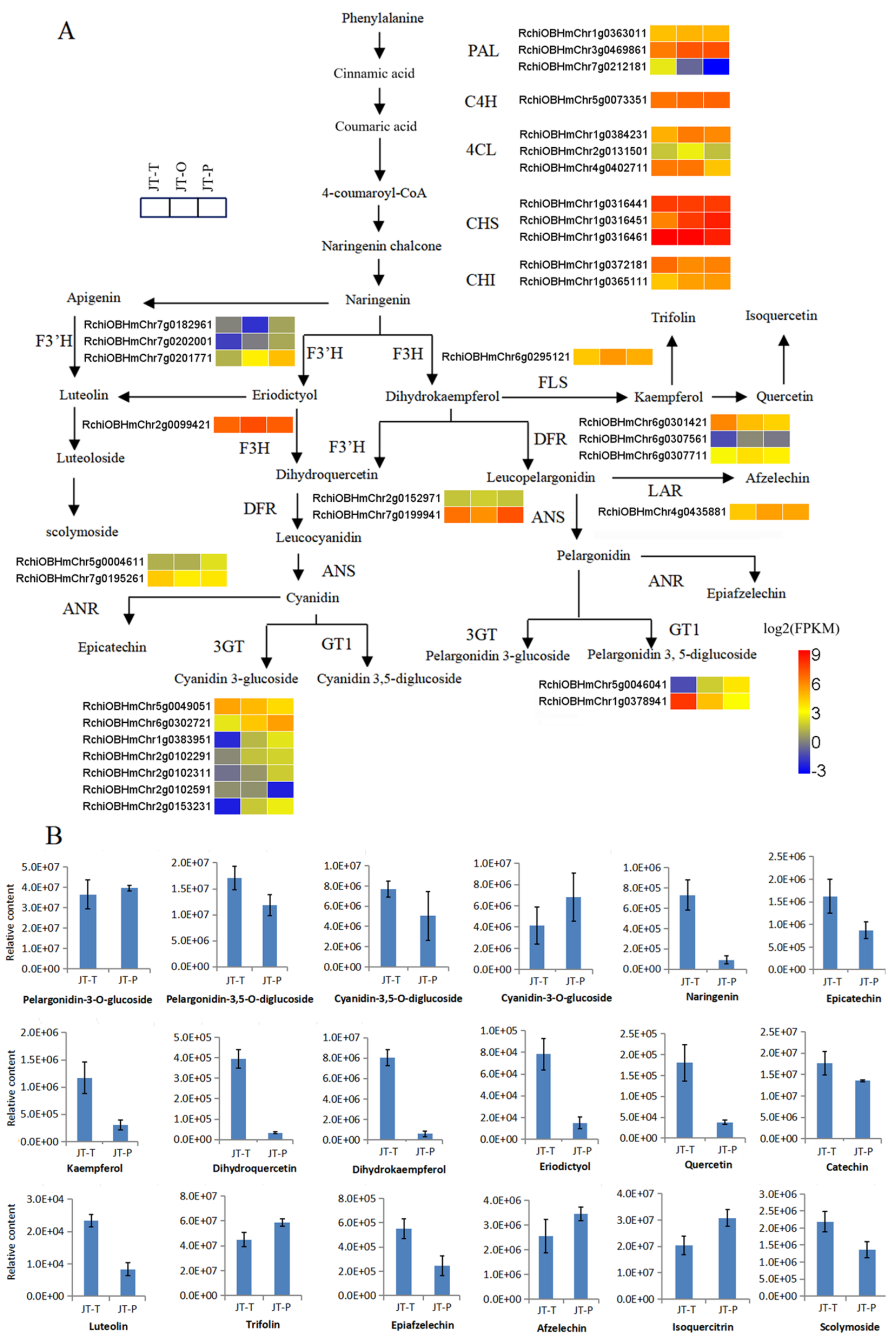
Biosynthetic pathway of flavonoids. **A**. Heatmaps illustrating the expression of genes related to the flavonoid biosynthetic pathway in rose petals. PAL, phenylalanine ammonialyase; C4H, cinnamate-4-hydroxylase; 4CL, 4-coumarate-CoA ligase; CHS, chalcone synthase; CHI, chalcone isomerase; F3H, flavanone 3-hydroxylase; F3’H, flavanone 3’-hydroxylase; FLS, favonol synthase; DFR, dihydroflavonol 4-reductase; ANS, anthocyanin synthase; ANR, anthocyanidin reductase; LAR, leucoanthocyanidin reductase; 3GT, anthocyanidin 3-O-glucosyltransfersae; GT1, anthocyanidin 5, 3-O-glucosyltransferase. The color scale indicates log2(FPKM) values. **B**. Flavonoids accumulation in JT-T and JT-P

There were 34 genes (25 DEGs) encoding 14 enzymes and four DAMs associated with the flavonoid biosynthetic pathway (Fig. 8, Tables S5, S8). These DAMs included dihydrokaempferol, pelargonidin-3,5-O-diglucoside (Pg3G5G), isoquercitrin, and trifolin. In the naringenin formation step, several DEGs were significantly down-expressed in the JT-T vs. JT-P comparison, such as *PAL* (*RchiOBHmChr7g0212181*), *4CL2* (*RchiOBHmChr4G0402711*), and *CHS* (*RchiOBHmChr1g0316461*). Naringenin was catalyzed by F3H to generate dihydrokaempferol. Compared with the JT-T stage, a notable decrease in dihydrokaempferol levels was detected in the petals at the JT-P stage. Meanwhile, there was a marked increase in the accumulation of trifolin and isoquercetin in the petals during this stage.

In addition, in the anthocyanin formation step, *GT1* (*RchiOBHmChr1g0378941*) and *DFR* (*RchiOBHmChr6g0301421*) were remarkably down-regulated in the petals at the JT-P stage compared to the JT-T stage. In this study, five types of anthocyanins including pelargonidin-3-O-glucoside, cyanidin3-O-glucoside, cyanidin-3,5-O-diglucoside, and pelargonidin-3-O-(6’’-O-malonyl) glucoside, and Pg3G5G, were identified from rose petals. Among these compounds, Pg3G5G was the only differentially accumulated anthocyanin that showed a significant reduction in the petals of the JT-P stage compared to the JT-T stage; however, this reduction was less than two-fold.

### Network analysis

Based on the aforementioned experimental results, it was noticed that the variation in carotenoid content emerged as the primary factor influencing the color variation of rose petals, followed by the variation in flavonoid content. To understand the regulation of the carotenoid and flavonoid pathways, the metabolic pathway-related genes, transcription factors, and metabolites were screened based on Pearson correlation analysis. The Pearson correlation analysis was conducted to examine the association between genes and metabolites, with a strong correlation defined as *P* < 0.05 and |r| > 0.8. The interactive networks among DEGs, DAMs, and TFs were constructed based on the results presented in Tables S9-S12.

**Fig. 9:**
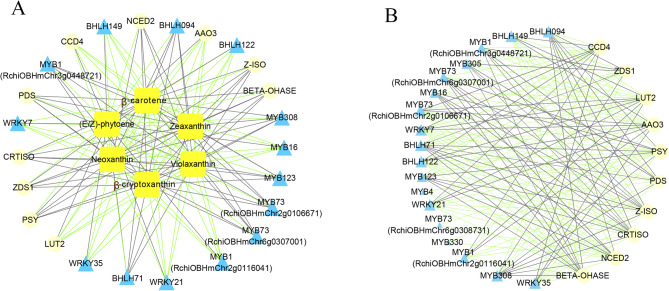
Network interaction diagrams of the carotenoid pathways in roses. **A**. Connection network between DEGs and DAMs. **B**. Connection network between TFs and carotenoid-related genes. Yellow squares represent carotenoid-related metabolites. Pale yellow circles represent carotenoid-related genes. Blue triangles represent TFs. Black lines and green lines represent positive and negative correlations, respectively, as determined by a Pearson correlation coefficient |r|> 0.8

According to the differential expression levels and annotation of TFs, as well as blast results, 18 *TFs* were chosen to investigate their correlations with the structural genes related to pigment metabolism (Table S13-S14). In the carotenoid pathway, the analysis revealed that 24 transcripts (10 carotenoid-related genes and 14 transcription factors) from the JT-T and JT-P stages exhibited significant correlation coefficients with the 6 carotenoid metabolites. As depicted in Fig. 9A, a network consisting of 30 nodes and 144 edges was constructed. All six DAMs in this carotenoid pathway network were identified as hub nodes, with 64 pairs exhibiting negative correlations and 80 pairs exhibiting positive correlations. Specifically, 7 *MYBs*, 4 *bHLHs*, and 3 *WRKYs* were strongly correlated with the 6 metabolites. Furthermore, *MYB308*, *MYB1* (*RchiOBHmChr3g0448721*), *MYB73*, *MYB123*, *BHLH71* and *BHLH094* were found to play positively correlated roles in this carotenoid pathway, while *BHLH149*, *MYB1* (*RchiOBHmChr2g0116041*), *MYB16*, *BHLH122*, *WRKY7*, *WRKY*21 and *WRKY35* showed negatively correlated roles. Additionally, *PSY*, *PDS*, *ZDS1*, *CRTISO*, *Z-ISO*, *NCED2* and *BETA-OHASE* showed positive correlations with β-carotenoid, β-cryptoxanthin, violaxanthin, zeaxanthin and neoxanthin but negative correlations with (E/Z)-phytoene. Conversely, *CCD4*, *LUT2* and *AAO3* exhibited positive correlations with (E/Z)-phytoene and negative correlations with the other metabolites.

Figure 9B revealed correlations between 18 transcription factors and 10 carotenoid-related structural genes across all three stages. As depicted in Fig. 9B, a network consisting of 28 nodes and 144 edges was constructed, with 77 pairs exhibiting a negative correlation and 67 pairs exhibiting a positive correlation. It was shown that 15 *TFs* had a strong correlation with multiple structural genes. Notably, *MYB308*, *BHLH71*, and *BHLH094* exhibited positive correlations with seven structural genes involved in the upstream pathway of carotenoid metabolism, while showing negative correlations with *CCD4*, *LUT2* and *AAO3* from the downstream pathway. Moreover, *MYB1* (*RchiOBHmChr3g0448721*) exhibited positive correlations with *PDS*, *ZDS1*, *Z-ISO*, *CRTISO*, and *BETA-OHASE*, while *MYB1* (*RchiOBHmChr2g0116041*) showed negative correlations with these genes. The findings suggest that these *TFs* may play pivotal roles in the regulation of carotenoid content. Additionally, *PSY*, *PDS*, *Z-ISO*, *CRTISO*, *ZDS1*, *AAO3*, *LUT2*, *BETA-OHASE*, *CCD4*, and *NCED2* displayed strong correlations with 15 *TFs* involved in the carotenoid pathway.

**Fig. 10:**
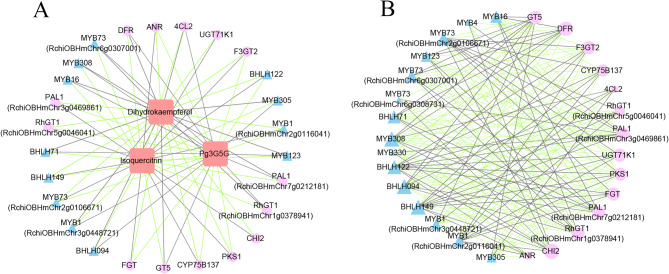
Network interaction diagrams of the flavonoid pathways in roses. **A**. Connection network between DEGs and DAMs. **B**. Connection network between TFs and flavonoid-related genes. Red squares represent flavonoid-related metabolites. Pink circles represent flavonoid-related genes. Blue triangles represent TFs. Black lines and green lines represent positive and negative correlations, respectively, as determined by a Pearson correlation coefficient |r|> 0.8

The diagram in Fig. 10A illustrates 26 transcripts (14 flavonoid biosynthesis-related genes and 12 transcription factors) and 3 DAMs (Dihydrokaempferol, Pg3G5G, and Isoquercitrin) from the JT-T and JT-P stages that exhibited strong correlations in the flavonoid biosynthetic pathway.

In Fig. 10A, it could be noticed that a network comprising 29 nodes and 76 edges was interconnected. The three DAMs in the flavonoid pathway network served as hub nodes, with 37 pairs exhibiting negative correlations and 39 pairs exhibiting positive correlations. Specifically, 8 *MYBs* and 4 *BHLHs* were strongly correlated with the 3 metabolites. Additionally, Fig. 10B revealed correlations between 15 transcription factors and 14 flavonoid-related structural genes from all three stages. As depicted in Fig. 10B, a network consisting of 29 nodes and 156 edges was constructed, with 86 pairs exhibiting a negative correlation and 70 pairs exhibiting a positive correlation. *MYB308*, *MYB16*, *MYB1* (*RchiOBHmChr2g0116041*, *RchiOBHmChr3g0448721*), *MYB73* (*RchiOBHmChr6g0307001*, *RchiOBHmChr2g0106671*), *BHLH094*, *BHLH149*, *BHLH122*, and *BHLH71* were strongly correlated with more than 10 flavonoid-related structural genes, respectively. Additionally, *PAL1*, *GT5*, *CHI2*, *DFR*, *FGT*, *PKS1*, *F3GT2*, *UGT71K1*, *RhGT1*, and *CYP75B137* exhibited significant correlations with multiple *TFs* in the flavonoid pathway.

## Discussion

### Metabolites in rose petals at two developmental stages

The present study employed ultra-performance liquid chromatography (UPLC) and mass spectrometry (MS)-based metabolomics approaches to investigate the metabolic composition of rose cultivar ‘Juicy Terrazza’ petals. A total of 872 primary and secondary metabolites were identified in rose petals. Some of the secondary metabolites included rosmarinic acid, mallotusinin, geraniin, canthaxanthin, echinenone, astaxanthin, and β-citraurin. These seven secondary metabolites have not been reported in these research reports [5, 14, 39–42]. Comparative analysis revealed significant differences in 153 accumulated metabolites (46 carotenoid metabolites and 107 non-carotenoid metabolites) between open flowers in JT-P and flower buds in JT-T. Specifically, 25 carotenoids and derivatives, 10 flavonoids, 9 tannins, 6 nucleotides and derivatives as well as 12 other metabolites were found to have significantly higher contents (≥ 2 fold) in the tangerine buds compared to the open flowers. Conversely, the levels of (E/Z)-phytoene, 11 amino acids and their derivatives, 6 alkaloids, and 19 other metabolites were significantly higher (≥ 2 fold) in the pink open flowers. Moreover, the contents of L-ascorbic acid and glutathione in JT-P flowers were exceptionally higher than those in JT-T buds, which can aid in the retardation of rose petal senescence.

Moreover, this study revealed a significant presence of carotenoids in the reddish-orange rose buds, which can play important roles in human health. They have been demonstrated to exert beneficial effects on antioxidant capacity, ocular health, cardiovascular well-being, cognitive function enhancement, and the reduction of certain cancer risks [43–46]. Furthermore, anthocyanins were also detected in the petals of this rose cultivar, which have beneficial effects on human health [47]. The aglycones of flavonols detected were kaempferol, quercetin, myricetin, isorhamnetin, herbacetin, and taxifolin. Additionally, studies have reported that kaempferol and quercetin are the primary aglycones of flavonols in the petals of rose species [5, 39, 48]. Moreover, flavonol glycosides are a crucial class of co-pigments that enhance the stability of flower color phenotype and possess significant biological activities [49, 50]. Kaempferol glycosides and quercetin glycosides have been studied for their pharmacological activities, such as antioxidant, anti-inflammatory, antiviral, anticancer, and cardio-protective properties [51–58]. In this particular study, it was found that kaempferol and quercetin remained the predominant aglycones of flavonols in the petals of the rose cultivar ‘Juicy Terrazza’. Additionally, this study identified an abundant kaempferol-7-O-glucoside in petals at JT-P, which has been demonstrated to have antiviral activity against HSV and HIV-1 infections [52, 53]. 22 triterpenes were discovered in our research, some of which exhibit anti-tumor, anti-inflammatory, and anti-HIV activities [59, 60]. To sum up, the rose petals possess a great variety of bioactive metabolites in this cultivar, especially the rose buds. Petals of ‘Juicy Terrazza’ may become candidates for anti-virus drug development. These research findings have well elucidated the chemical compositions of rose petals and deepened our understanding of rose metabolites.

### Carotenoid regulation in rose petals

Geranylgeranyl diphosphate (GGPP) serves as a direct precursor for carotenoid biosynthesis, wherein two GGPP molecules undergo condensation to produce the colorless phytoene via the action of phytoene synthase (PSY). PSY, functioning as a core enzyme, plays a pivotal role in determining the overall accumulation of carotenoids in plant tissues and acts as a rate-limiting factor in the carotenoid metabolic pathway [61, 62]. A common phytoene synthase mutation results in the inability to synthesize carotenoids in the white petals of the California poppy [63]. During the ripening process of tomato fruits, the expression levels of genes including *PSY*, *PDS*, *ZDS*, and *CRTISO* progressively increase, resulting in the substantial accumulation of the red pigment lycopene in the fruit [64]. In our research, *PSY* (*RchiOBHmChr2g0152351*) exhibited significant downregulation in JT-T vs. JT-P. This finding was consistent with the trend of reduced accumulation of β-carotene, violaxanthin, zeaxanthin, β-cryptoxanthin and neoxanthin; however, it was opposite to the accumulation of (E/Z)-phytoene. We found that the expression of the *PSY*, *PDS*, *ZISO*, and *ZDS* genes was remarkably reduced in JT-P compared with JT-O. To a significant extent, the decreased expression of these genes was the immediate cause of the reduction in carotenoid contents during the JT-P stage.

As a central metabolite, β-carotene exhibited strong correlations with multiple DEGs. It accumulated prominently in the petals at the JT-T stage, whereas only a small amount was detected in the petals at the JT-P stage. Carotenoid cleavage dioxygenase (CCD) is a crucial enzyme in the carotenoid metabolic pathway in plants and can catalyze the cleavage of carotenoids. Previous studies showed that *OfCCD4* was strongly expressed in ‘Yingui’ throughout the flowering process, while it was hardly expressed in ‘Dangui’ with orange-red petals [65]. The positive regulatory factor *OfWRKY3*, which acted as a positive regulator of *OfCCD4* in Osmanthus, promoted carotenoid degradation and led to a light hue [65]. Ureshino’s research demonstrated that the high expression level of *CCD4* in azalea petals was responsible for reducing carotenoid content in the progeny [66]. The increased carotenoid content observed in yellow petunia flowers can be attributed to the absence of *CCD4* expression, and transformed CCD4 expression leads to a decrease in carotenoid levels [67]. Our study results revealed that *RcCCD4* exhibited strong expression in the JT-O and JT-P stages, but was rarely expressed in the JT-T stage. This result suggested that the high expression of *RcCCD4* in the petals of open flowers predominantly regulates the reduction of β-carotene.

Moreover, previous studies have indicated that transcription factors can regulate carotenoid pigmentation. Toledo-Ortiz et al.'s research revealed that the transcription factor *PIF1* directly binds to the G-Box element on the *PSY* promoter region, thereby inhibiting *PSY* transcription and consequently reducing carotenoid content [68]. Additionally, an R2R3-MYB protein (WP1) associated with anthocyanin in *Medicago truncatula* has a crucial function in the regulation of carotenoid pigmentation in flowers [22]. Repression of carotenoid biosynthesis genes and reduction of the level of carotenoids in *M. lewisii* flowers are observed as a consequence of the presence of loss-of-function mutations in *RCP1* (*R2R3-MYB*) [69]. Additionally, *RcMYB1* (*RchiOBHmChr3g0492711*) in roses has been demonstrated to play a role in the regulation of carotenoid metabolism [27]. In this study, the correlation analysis revealed that 7 *MYBs*, 4 *bHLHs* and 3 *WRKYs*, especially *MYB308*, *MYB1* (*RchiOBHmChr3g0448721*, *RchiOBHmChr2g0116041*), *BHLH71*, *BHLH094*, *WRKY35* and *WRKY7*, were strongly correlated with carotenoid metabolism. We found that *MYB308* and *MYB1* (*RchiOBHmChr3g0448721*) were highly expressed in the reddish-orange bud, and their expression gradually decreased as the flower color changed, whereas *MYB1* (*RchiOBHmChr2g0116041*) exhibited the opposite trend. *MYB308 and MYB1* (*RchiOBHmChr2g0116041*, *RchiOBHmChr3g0448721*) displayed high similarity to *AtMYB4*, *AtMYB113* and *AtMYB114*, respectively, which were involved in the regulation of pigment biosynthesis [70, 71]. Therefore, these three *TFs* could play crucial roles in regulating the transition of flower color.

### Flavonoid regulation in rose petals

Flavonoids, as the main secondary metabolites in rose petals, play a crucial role in influencing floral coloration, particularly various anthocyanins. Flavones and flavonols, which act as co-pigments for anthocyanins, are nearly colorless or pale yellow compounds [3, 72, 73]. The biosynthesis of anthocyanin glycosides generally proceeds through three stages: First, phenylalanine is enzymatically converted into coumaroyl-CoA. Second, coumaroyl-CoA is further converted to form dihydroflavonol. Finally, during the third stage, various anthocyanidins are synthesized. The synthesis pathway of rose anthocyanin glycosides primarily involves the expression of structural genes that encode crucial enzymes responsible for anthocyanin biosynthesis, including *PAL*, *4CL*, *CHS*, *CHI*, *F3H*, *F3’H*, *DFR*, *FLS*, *ANS*, *GT1*, and *3GT*. However, there is still a lack of comprehensive documentation in roses regarding various transcription factors and structural genes that control the anthocyanin pathways [42].

The flavonoid glycosylhydrolase enzyme serves as a crucial hub in the flavonoid metabolic pathway, competing with dihydroflavonol 4-reductase for the shared substrate dihydroflavonol [74]. When the predominant flow of flavonoid metabolism is directed towards quercetin synthesis, it exerts a significant influence on anthocyanidin synthesis, leading to reduced accumulation of anthocyanidins [75]. The overexpression of Rosa *RrFLS1*, peach *PpFLS*, tea *CsFLS*, and chrysanthemum *CmFLS* in tobacco resulted in a significant increase in flavonoid content and a lighter hue of the flower corolla, while concurrently leading to a marked reduction in anthocyanidin levels [76–78]. Additionally, F3’H catalyzes the hydroxylation of dihydrokaempferol to form dihydroquercetin [3]. In this study, the dihydrokaempferol metabolite in the petals was significantly reduced, exhibiting a thirteen-fold decrease in JT-P compared to JT-T. Therefore, we hypothesize that the significant downregulation of structural genes *4CL2* (*RchiOBHmChr4g0402711*) and *PAL1* (*RchiOBHmChr7g0212181*), along with the remarkable up-regulation of structural genes *F3’H* (*RchiOBHmChr7g0201771*) and *FLS* (*RchiOBHmChr6g0295121*), may directly contribute to the reduction in the dihydrokaempferol metabolite.

Moreover, we identified five types of anthocyanins in the rose petals. These anthocyanins serve as the primary pigments responsible for the pink petal coloration during the JT-P stage. To some extent, compared with the petals of the JT-T stage, a decrease was observed in Pg3G5G content within the petals of the JT-P stage. UFGT is responsible for converting unstable anthocyanidins into stable anthocyanins in the anthocyanin synthesis pathway. The genes, such as *ANS*, *CHS*, *DFR*, and *UFGT* exhibit high expression levels in the red petals of variegated peach flowers. In contrast, in the white petals, *ANS*, *CHS*, and *DFR* are down-regulated, while UFGT exhibits only minimal expression [79]. In red and white picotee bicolor petals of the lotus, the defect in NnUFGTs accumulation resulted in the failure of anthocyanidin glycosylation in the white part of the petals [80]. Our research found that the expression levels of *GT1* (*RchiOBHmChr1g0378941*) and *DFR* (*RchiOBHmChr6g0301421*) were significantly down-regulated in JT-P, which may lead to a substantial decrease in Pg3G5G levels.

Furthermore, a prior study has indicated that *RcMYB114* was involved in the regulation of anthocyanin biosynthesis in roses [81]. Moreover, the overexpression of *RrMYB5* and *RrMYB10* led to an increased accumulation of proanthocyanidins in both *R. rugosa* and tobacco [82]. Additionally, *RrMYB108*, *RrMYB114*, and *RrC1* may play crucial roles in regulating the petal color in *R. rugosa* [83]. Our research showed that the expression levels of the 14 structural genes and *TFs* (11 *MYBs*, 4 *bHLHs*) were strongly correlated with the content of dihydrokaempferol and Pg3G5G (Fig. 10A). Notably, 11 *TFs* were strongly related to more than ten structural genes. Among these, *MYB308* and *MYB1 (RchiOBHmChr3g0448721*, *RchiOBHmChr2g0116041)* showed high similarity to *AtMYB4*, *AtMYB113*, and *AtMYB114*. Therefore, we hypothesize that these three transcription factors could be involved in the regulation of flavonoid metabolism in rose petals.

Previous studies have demonstrated that MYB, bHLH, and WDR transcription factor families typically form MBW (MYB-bHLH-WDR) protein complexes to cooperatively regulate anthocyanin synthesis [21], and additionally play critical roles in regulating the accumulation of carotenoids [22, 23]. Specifically, MYB transcription factors function as key regulators in the biosynthesis of anthocyanins and the metabolism of carotenoids [20–23, 25–27]. Our research revealed that during the color transition of rose petals, the expression levels of *MYB308* and *MYB1* (*RchiOBHmChr3g0448721*, *RchiOBHmChr2g0116041*) showed significant variation. Furthermore, they showed strong correlations with multiple structural genes involved in the biosynthesis of carotenoids and anthocyanins. Therefore, these *MYBs* may play crucial roles in regulating pigment metabolism in this rose cultivar.

In this study, transcriptomic analysis was conducted on three developmental stages of rose petals (JT-T, JT-O, JT-P), but metabolomic analysis only covered two stages (JT-T and JT-P). This resulted in an inability to fully synchronize the association between transcriptome and metabolome changes. So, the construction of the relationship between genes and metabolites may be subject to certain deviations. Future research could conduct a comprehensive analysis of the molecular regulatory network underlying the color transformation process in rose petals by synchronously examining transcriptome, metabolome, and proteome data obtained from the three developmental stage.

## Conclusions

Our research findings indicate that the petal color of the rose cultivar ‘Juicy Terrazza’ is primarily influenced by the concentrations of carotenoids and anthocyanins. Notably, the degradation of carotenoids, particularly violaxanthin and its derivatives, as well as β-carotene, plays a more significant role in influencing changes in petal coloration in roses compared to anthocyanins. The contents of Pg3G5G and other flavonoids in the rose petals have a secondary effect on petal color change. The key structural genes as well as the major transcription factors participating in the carotenoid and anthocyanin synthesis pathways regulate carotenoid and anthocyanin contents during flower color changes. In this study, we provided new insights into the metabolic mechanisms of flavonoids and carotenoids in roses through the integrative analysis of transcriptomic and metabolomic data. These findings establish a groundwork for further investigations into the mechanisms of color transition in rose petals, and provide valuable information for molecular breeding of ornamental plants with specific flower colors.

## Electronic supplementary material

Below is the link to the electronic supplementary material.

Supplementary Material 1

## Data Availability

The RNA-seq datasets generated during the study are available in the NCBI SRA repository under accession number PRJNA1259577.

## Abbreviations

PSY: Phytoene synthase
PDS: Phytoene desaturase
ZISO: Zeta-carotene isomerase
ZDS: Zeta-carotene desaturase
crtISO: Carotenoid isomerase
lcyB: Lycopene beta cyclase
lcyE: Lycopene epsilon cyclase
LUT5: Beta-ring hydroxylase
HYb: Beta-carotene hydroxylase
HYe: Carotenoid epsilon hydroxylase
VDE: Violaxanthin de-epoxidase
ZEP: Zeaxanthin epoxidase
NXS: Neoxanthin synthase
D27: Beta-carotene isomerase
CCD: Carotenoid cleavage dioxygenase
CCD8B: Carlactone synthase
ABA2: Xanthoxin dehydrogenase
AAO3: Indole-3-acetaldehy-deoxidase
PAL: Phenylalanine ammonialyase
C4H: Cinnamate − 4-hydroxylase
4CL: 4-coumarate-CoA ligase
CHS: Chalcone synthase
CHI: Chalcone isomerase
F3H: Flavanone 3-hydroxylase
F3’H: Flavanone 3’-hydroxylase
FLS: Favonol synthase
DFR: Dihydroflavonol 4-reductase
ANS: Anthocyanin synthase
ANR: Anthocyanidin reductase
LAR: Leucoantho cyanidin reductase
3GT: Anthocyanidin 3-O-glucosyltransfersae
GT1: Anthocyanidin 5, 3-O-glucosyltransferase
Pg3G5G: Pelargonidin 3, 5-diglucoside
