# Supplementary material for: Integrated transcriptome and metabolome analyses provide molecular insights into the transition of flower color in the rose cultivar ‘Juicy Terrazza’
Source: BMC Plant Biol. 2025 Jul 4;25:883. doi: 10.1186/s12870-025-06794-2 (PMC12231300; doi:10.1186/s12870-025-06794-2)
Supplement: Supplementary file 1 — Supplementary Material 1 [file 12870_2025_6794_MOESM1_ESM.docx]

Supplementary information

Table S1. Overview of the transcriptome sequencing data obtained using illumina technology.

| **Sample** | **Raw Reads** | **Clean Reads** | **Raw Data (bp)** | **Clean Data (bp)** | **Q20 (%)** | **Q30 (%)** | **GC (%)** |
| --- | --- | --- | --- | --- | --- | --- | --- |
| JT-T-1 | 57618086 | 57405578 (99.63%) | 8642712900 | 8552985160 | 97.21 | 92.29 | 45.68 |
| JT-T-2 | 63326508 | 63111758 (99.66%) | 9498976200 | 9406610635 | 97.29 | 92.49 | 45.66 |
| JT-T-3 | 57065160 | 56828300 (99.58%) | 8559774000 | 8467567338 | 97.01 | 91.84 | 45.68 |
| JT-O-1 | 49369584 | 49242214 (99.74%) | 7405437600 | 7342185097 | 97.51 | 92.85 | 45.48 |
| JT-O-2 | 53611220 | 53444528 (99.69%) | 8041683000 | 7973235907 | 97.25 | 92.38 | 45.54 |
| JT-O-3 | 66711678 | 66447702 (99.60%) | 10006751700 | 9911373949 | 97.11 | 92.06 | 45.48 |
| JT-P-1 | 40450038 | 40308242 (99.65%) | 6067505700 | 6000028502 | 97.47 | 92.70 | 45.56 |
| JT-P-2 | 62221948 | 61995038 (99.64%) | 9333292200 | 9238568138 | 97.24 | 92.33 | 45.57 |
| JT-P-3 | 54540884 | 54336052 (99.62%) | 8181132600 | 8102038428 | 97.32 | 92.53 | 45.53 |

Table S2. Number of reads of the nine samples mapped to reference sequences.

| **Sample** | **Total** | **Unique_Mapped (%)** | **Multiple_Mapped (%)** | **Total_Mapped (%)** |  |
| --- | --- | --- | --- | --- | --- |
| JT-T-1 | 57362310 | 47842946 (83.40%) | 1685937 (2.94%) | 49528883 (86.34%) | |
| JT-T-2 | 63068558 | 52697078 (83.56%) | 1852618 (2.94%) | 54549696 (86.49%) | |
| JT-T-3 | 56793140 | 47323381 (83.33%) | 1644425 (2.90%) | 48967806 (86.22%) | |
| JT-O-1 | 49209062 | 41002040 (83.32%) | 1491542 (3.03%) | 42493582 (86.35%) | |
| JT-O-2 | 53399266 | 44083532 (82.55%) | 1609277 (3.01%) | 45692809 (85.57%) | |
| JT-O-3 | 66403612 | 54855122 (82.61%) | 2014696 (3.03%) | 56869818 (85.64%) | |
| JT-P-1 | 40274456 | 33561642 (83.33%) | 1088030 (2.70%) | 34649672 (86.03%) | |
| JT-P-2 | 61901160 | 51258082 (82.81%) | 1689242 (2.73%) | 52947324 (85.54%) | |
| JT-P-3 | 57362310 | 45006751 (82.90%) | 1478000 (2.72%) | 46484751 (85.63%) | |


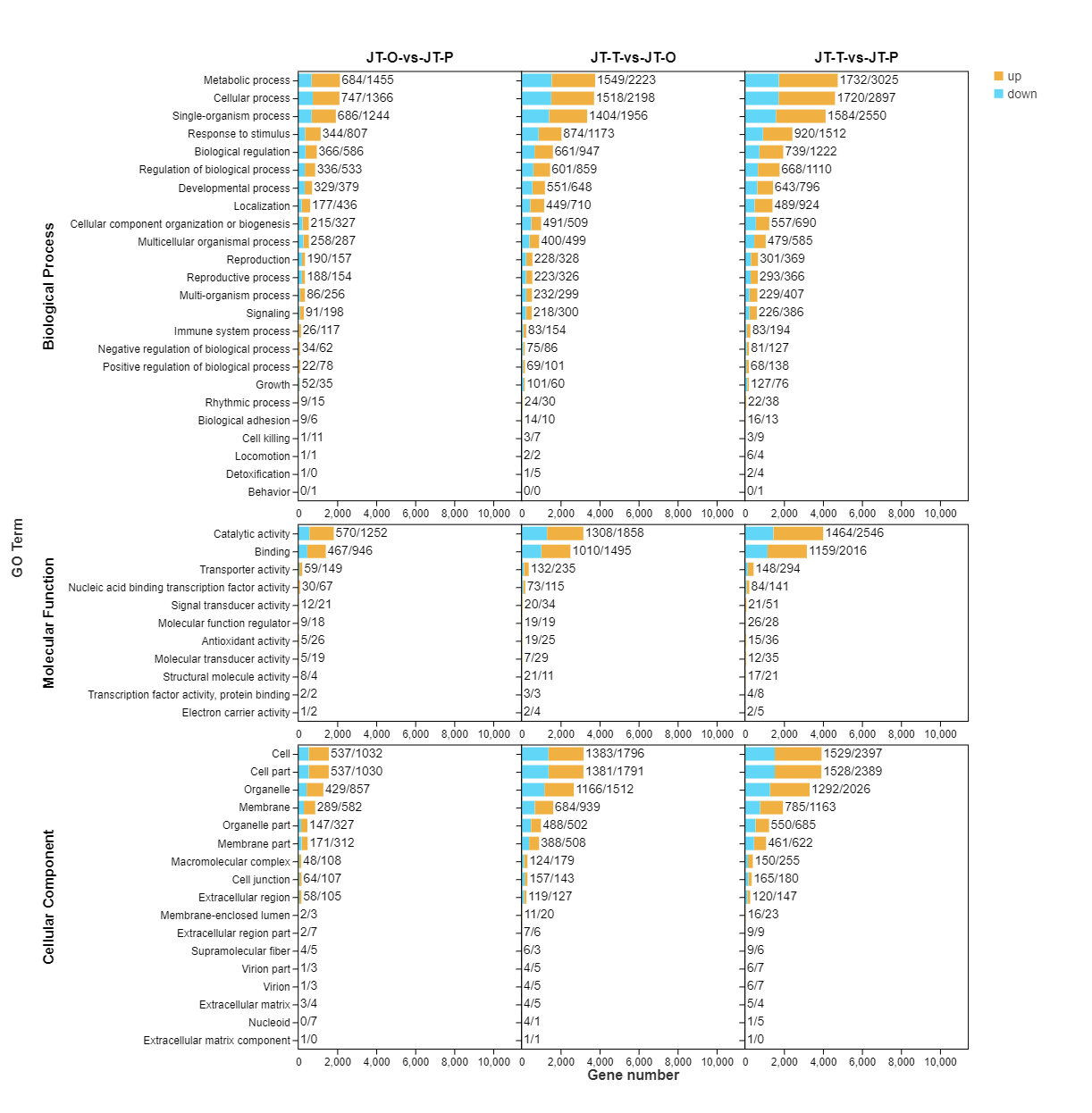


Figure S1. GO classification of DEGs. GO significant enrichment analysis of up- and down- regulated DEGs between the three comparisons: JT-O vs. JT-P, JT-T vs. JT-O and JT-T vs. JT-P.

Table S3. The FPKM values of the expression level of *TFs* (*MYB*, *bHLH*, *AP2/EREBP*, *WRKY* and *WDR*) in roses.

| **TFs** | **Genes** | **JT-T** | **JT-O** | **JT-P** |
| --- | --- | --- | --- | --- |
| *MYB* | RchiOBHmChr2g0116041 | 0.54 | 32.49 | 114.60 |
|  | RchiOBHmChr3g0448721 | 730.36 | 551.71 | 64.06 |
|  | RchiOBHmChr7g0186441 | 0.41 | 3.90 | 3.08 |
|  | RchiOBHmChr3g0492711 | 12.68 | 39.58 | 42.61 |
|  | RchiOBHmChr6g0304811 | 197.24 | 3.74 | 4.57 |
|  | RchiOBHmChr2g0167441 | 55.81 | 19.21 | 15.36 |
|  | RchiOBHmChr1g0354061 | 0.71 | 7.08 | 4.06 |
|  | RchiOBHmChr6g0311741 | 5.59 | 1.40 | 0.14 |
|  | RchiOBHmChr3g0491061 | 4.36 | 1.24 | 2.75 |
|  | RchiOBHmChr7g0178691 | 1.76 | 5.73 | 1.48 |
|  | RchiOBHmChr2g0172331 | 390.49 | 347.33 | 76.29 |
|  | RchiOBHmChr7g0181361 | 18.93 | 6.01 | 4.02 |
|  | RchiOBHmChr6g0252211 | 98.83 | 54.76 | 14.67 |
|  | RchiOBHmChr7g0178681 | 17.52 | 50.40 | 19.79 |
|  | RchiOBHmChr7g0228621 | 37.94 | 98.38 | 76.31 |
|  | RchiOBHmChr4g0441081 | 68.90 | 192.95 | 144.23 |
|  | RchiOBHmChr2g0106671 | 22.11 | 54.21 | 70.80 |
|  | RchiOBHmChr6g0255341 | 2.17 | 3.90 | 0.74 |
|  | RchiOBHmChr6g0307001 | 42.06 | 5.53 | 3.30 |
|  | RchiOBHmChr6g0308731 | 72.94 | 221.34 | 157.05 |
|  | RchiOBHmChr2g0116071 | 8.26 | 8.63 | 2.95 |
|  | RchiOBHmChr4g0426181 | 0.68 | 2.72 | 16.17 |
| *bHLH* | RchiOBHmChr6g0288541 | 11.81 | 2.52 | 2.62 |
|  | RchiOBHmChr2g0109611 | 1.16 | 0.31 | 1.33 |
|  | RchiOBHmChr2g0109621 | 1.26 | 0.28 | 0.74 |
|  | RchiOBHmChr5g0010631 | 1.62 | 6.10 | 12.95 |
|  | RchiOBHmChr4g0399211 | 2.47 | 3.83 | 9.75 |
|  | RchiOBHmChr6g0283511 | 0.99 | 1.57 | 0.23 |
|  | RchiOBHmChr2g0085911 | 2.13 | 8.13 | 14.92 |
|  | RchiOBHmChr6g0291401 | 0.21 | 4.57 | 6.80 |
|  | RchiOBHmChr7g0210101 | 147.78 | 57.62 | 9.76 |
|  | RchiOBHmChr4g0445091 | 330.98 | 214.50 | 56.16 |
|  | RchiOBHmChr7g0180121 | 167.05 | 458.76 | 185.75 |
|  | RchiOBHmChr1g0337011 | 1.22 | 17.80 | 10.39 |
|  | RchiOBHmChr6g0245181 | 14.37 | 6.26 | 8.71 |
|  | RchiOBHmChr5g0077341 | 4.23 | 20.55 | 33.96 |
|  | RchiOBHmChr7g0187261 | 23.98 | 0.15 | 0.12 |
|  | RchiOBHmChr4g0425781 | 10.72 | 23.55 | 10.78 |
|  | RchiOBHmChr3g0457291 | 9.95 | 33.86 | 66.72 |
|  | RchiOBHmChr4g0405961 | 1.70 | 5.87 | 8.90 |
|  | RchiOBHmChr1g0365881 | 0.67 | 2.97 | 6.27 |
|  | RchiOBHmChr7g0182341 | 0.07 | 4.09 | 8.26 |

Continued

| *WDR* | RchiOBHmChr2g0109631 | 5.12 | 12.49 | 17.98 |
| --- | --- | --- | --- | --- |
|  | RchiOBHmChr2g0120151 | 2.39 | 0.55 | 0.36 |
|  | RchiOBHmChr2g0165901 | 5.16 | 13.27 | 19.34 |
|  | RchiOBHmChr3g0470921 | 3.50 | 11.11 | 11.76 |
|  | RchiOBHmChr5g0036881 | 4.25 | 11.49 | 9.48 |
|  | RchiOBHmChr7g0224541 | 1.53 | 4.22 | 4.18 |
|  | RchiOBHmChr6g0290561 | 3.52 | 9.42 | 12.94 |
|  | RchiOBHmChr7g0189251 | 1.28 | 2.98 | 4.12 |
|  | RchiOBHmChr7g0193241 | 0.45 | 1.47 | 2.48 |
| *WRKY* | RchiOBHmChr1g0359091 | 2.51 | 8.62 | 20.35 |
|  | RchiOBHmChr1g0372521 | 1.09 | 0.90 | 3.09 |
|  | RchiOBHmChr1g0380121 | 0.01 | 0.92 | 3.68 |
|  | RchiOBHmChr2g0106361 | 4.18 | 26.33 | 42.51 |
|  | RchiOBHmChr2g0133001 | 4.67 | 8.44 | 13.63 |
|  | RchiOBHmChr3g0450591 | 0.02 | 3.27 | 5.75 |
|  | RchiOBHmChr3g0460361 | 1.48 | 0.08 | 3.41 |
|  | RchiOBHmChr3g0461481 | 3.81 | 31.67 | 47.00 |
|  | RchiOBHmChr4g0398741 | 3.21 | 10.85 | 6.06 |
|  | RchiOBHmChr4g0425801 | 0.27 | 1.13 | 5.45 |
|  | RchiOBHmChr4g0429851 | 0.10 | 3.18 | 14.31 |
|  | RchiOBHmChr4g0439041 | 3.35 | 16.57 | 18.79 |
|  | RchiOBHmChr5g0002561 | 0.63 | 5.24 | 27.56 |
|  | RchiOBHmChr5g0011581 | 0.65 | 1.58 | 4.17 |
|  | RchiOBHmChr5g0071811 | 0.28 | 3.19 | 7.55 |
|  | RchiOBHmChr6g0299501 | 0.08 | 1.04 | 4.16 |
|  | RchiOBHmChr6g0308491 | 1.35 | 3.54 | 2.39 |
|  | RchiOBHmChr7g0195191 | 6.26 | 8.07 | 22.44 |
|  | RchiOBHmChr7g0202671 | 0.20 | 19.09 | 62.14 |
|  | RchiOBHmChr7g0241021 | 6.54 | 61.85 | 88.87 |
| *AP2/EREBP* | RchiOBHmChr6g0299771 | 1.63 | 12.78 | 14.01 |
|  | RchiOBHmChr6g0295481 | 1.76 | 1.03 | 5.07 |
|  | RchiOBHmChr2g0095581 | 25.64 | 0.24 | 0.16 |
|  | RchiOBHmChr1g0360021 | 22.97 | 25.23 | 2.05 |
|  | RchiOBHmChr2g0115041 | 19.15 | 2.66 | 14.60 |
|  | RchiOBHmChr7g0195031 | 5.04 | 10.46 | 26.07 |
|  | RchiOBHmChr6g0294441 | 9.72 | 0.52 | 1.89 |
|  | RchiOBHmChr1g0373641 | 18.15 | 324.98 | 276.54 |
|  | RchiOBHmChr6g0257181 | 10.68 | 0.32 | 0.10 |
|  | RchiOBHmChr6g0274591 | 0.15 | 7.33 | 10.34 |
|  | RchiOBHmChr4g0428891 | 4.20 | 25.59 | 48.74 |

Continued

| *AP2/EREBP* | RchiOBHmChr5g0008971 | 0.73 | 1.39 | 5.29 |
| --- | --- | --- | --- | --- |
|  | RchiOBHmChr7g0204641 | 4.94 | 1.89 | 3.18 |
|  | RchiOBHmChr1g0373621 | 16.73 | 80.57 | 77.36 |
|  | RchiOBHmChr5g0041261 | 13.24 | 9.41 | 13.49 |
|  | RchiOBHmChr5g0008991 | 59.11 | 22.74 | 27.63 |
|  | RchiOBHmChr1g0373631 | 56.35 | 155.40 | 164.96 |
|  | RchiOBHmChr7g0204611 | 14.50 | 7.67 | 6.73 |
|  | RchiOBHmChr2g0106221 | 26.52 | 63.70 | 49.58 |
|  | RchiOBHmChr1g0376641 | 8.00 | 2.32 | 2.15 |
|  | RchiOBHmChr2g0118211 | 0.96 | 13.96 | 19.51 |
|  | RchiOBHmChr6g0298011 | 120.45 | 20.83 | 4.43 |
|  | RchiOBHmChr6g0308371 | 23.37 | 98.67 | 132.95 |
|  | RchiOBHmChr2g0135921 | 83.51 | 0.97 | 0.08 |
|  | RchiOBHmChr5g0061491 | 8.01 | 22.95 | 24.45 |
|  | RchiOBHmChr5g0061501 | 5.58 | 14.99 | 16.88 |


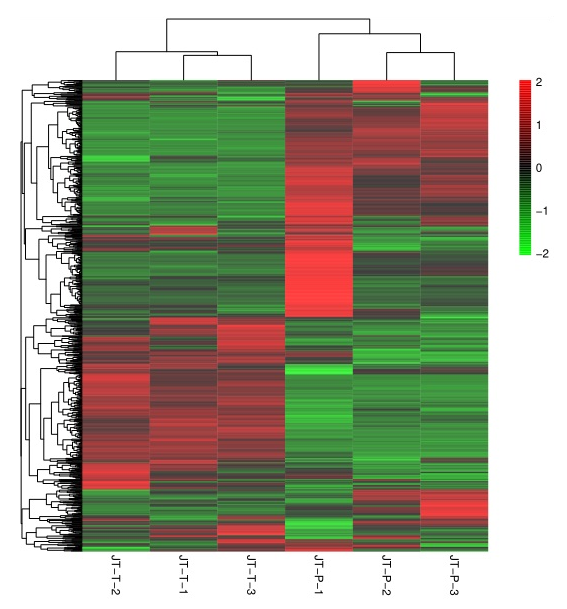


Figure S2. A heatmap of the 823 metabolites from rose petals of the two stages (JT-T and JT-P). The color scale (from red to green) indicated normalized metabolite contents using the row Z-score..

Table S4. Functional categories of the identified metabolites in rose petals.

| **Type** | **Number** | **Percentage** |
| --- | --- | --- |
| all | 823 | 100 |
| Phenolic acids | 127 | 15.431 |
| Organic acids | 74 | 8.991 |
| Flavonols | 69 | 8.384 |
| Amino acids and derivatives | 68 | 8.262 |
| Flavonoid | 57 | 6.926 |
| Free fatty acids | 54 | 6.561 |
| Saccharides and Alcohols | 51 | 6.197 |
| Nucleotides and derivatives | 47 | 5.711 |
| Tannin | 44 | 5.346 |
| LPC(lysophosphatidylcholine) | 33 | 4.01 |
| Alkaloids | 20 | 2.43 |
| Triterpene | 20 | 2.43 |
| Flavanols | 17 | 2.066 |
| Glycerol ester | 16 | 1.944 |
| Vitamin | 16 | 1.944 |
| LPE (lysophosphatidyl ethanolamine) | 15 | 1.823 |
| Phenolamine | 13 | 1.58 |
| Lignans | 11 | 1.337 |
| Dihydroflavone | 8 | 0.972 |
| Others | 8 | 0.972 |
| Chalcones | 7 | 0.851 |
| Flavonoid carbonoside | 7 | 0.851 |
| Plumerane | 7 | 0.851 |
| Proanthocyanidins | 7 | 0.851 |
| Dihydroflavonol | 6 | 0.729 |
| Anthocyanins | 5 | 0.608 |
| Coumarins | 4 | 0.486 |
| Monoterpenoids | 3 | 0.365 |
| Ditepenoids | 2 | 0.243 |
| Sesquiterpenoids | 2 | 0.243 |
| Triterpene Saponin | 2 | 0.243 |
| Isoflavones | 1 | 0.122 |
| PC (phosphatidylcholine) | 1 | 0.122 |
| Sphingolipids | 1 | 0.122 |

Table S5. Content comparisons of compounds extracted from rose petals at two stages (JT-P and JT-T).

| **Compound** | **Class** | **log2_FC (JT-P / JT-T)** |
| --- | --- | --- |
| Luteolin-3'-O-glucoside | Flavonoid | -0.22 |
| Aromadendrin-7-O-glucoside | Flavonoid | 0.92 |
| Orientin-2''-O-xyloside | Flavonoid | -0.69 |
| Luteolin-7-O-(6''-caffeoyl) rhamnoside | Flavonoid | -0.44 |
| Luteolin-7-O-neohesperidoside (Lonicerin) | Flavonoid | -0.60 |
| Kaempferol-3-O-(6''-galloyl) glucoside | Flavonoid | -0.60 |
| Kaempferol-7-O-glucoside | Flavonols | 0.37 |
| Kaempferol-3-O-galactoside (Trifolin) | Flavonols | 0.38 |
| Quercetin-3-O-galactoside (Hyperin) | Flavonols | 0.46 |
| Quercetin-4'-O-glucoside (Spiraeoside) | Flavonols | 0.68 |
| Quercetin-3-O-(4''-O-galloyl) arabinoside | Flavonols | -2.38 |
| Kaempferol-3-O-neohesperidoside | Flavonols | -0.69 |
| Kaempferol-3-O-glucoside-7-O-rhamnoside | Flavonols | -0.69 |
| Quercetin-3-O-(6''-p-Coumaroyl) glucoside | Flavonols | -1.25 |
| Quercetin-3-O-(6''-p-Coumaroyl) galactoside | Flavonols | -1.30 |
| Quercetin-7-O-rutinoside | Flavonols | -1.22 |
| Quercetin-3-O-(4''-O-glucosyl)rhamnoside | Flavonols | -1.14 |
| Quercetin-3-O-neohesperidoside | Flavonols | -1.09 |
| Kaempferol-3-O-(2''-O-galloyl) glucuronide | Flavonols | -0.78 |
| Quercetin-3-O-(6''-galloyl) glucoside | Flavonols  Flavonols  Flavonols  Flavonols  Flavonols  Flavonols  Flavonols  Flavonols  Flavonols | -1.13 |
| Quercetin-3-O-(6''-galloyl) galactoside | Flavonols  Flavonols  Flavonols  Flavonols  Flavonols  Flavonols  Flavonols  Flavonols  Flavonols | -1.00 |
| Quercetin-3-O-(2'',3''-digalloyl)-glucoside | Flavonols  Flavonols  Flavonols  Flavonols  Flavonols  Flavonols  Flavonols  Flavonols  Flavonols | -3.08 |
| Quercetin-3-O-glucoside (Isoquercitrin) | Flavonols  Flavonols  Flavonols  Flavonols  Flavonols  Flavonols  Flavonols  Flavonols  Flavonols | 0.60 |
| Dihydrokaempferol (Aromadendrin) | Dihydroflavonol | -3.79 |
| Pelargonidin-3,5-O-diglucoside | Anthocyanins | -0.53 |
| Epicatechin glucoside | Flavanols | 2.53 |
| 1-O-Vanilloyl-D-Glucose | Phenolic acids | 3.51 |
| 3-Hydroxy-5-Methylphenol-1-O-(6'-Galloyl) Glucoside | Phenolic acids | 8.96 |
| 3-O-Digalloyl quinic acid | Phenolic acids | -2.66 |
| p-Coumaroylquinic acid-4'-O-glucuronide | Phenolic acids | -2.27 |
| 4-Nitrophenol | Phenolic acids | 3.01 |
| 2-Hydroxycinnamic acid; O-Coumaric Acid | Phenolic acids | -4.91 |
| Protocatechuic Acid Methyl Ester | Phenolic acids | 1.89 |
| Diisobutyl phthalate | Phenolic acids | 0.26 |
| Dibutyl phthalate | Phenolic acids | 0.29 |
| 3-Hydroxy-5-Methylphenol-1-O-Glucoside | Phenolic acids | 5.24 |
| Brevifolin carboxylic acid | Phenolic acids | -0.53 |
| 5-O-Galloylshikimic acid | Phenolic acids | -1.20 |
| 3-O-Galloylshikimic acid | Phenolic acids | -1.36 |
| Piperidine | Alkaloids | 2.67 |
| Choline | Alkaloids | -0.37 |
| Nicotinic Acid Methyl Ester | Alkaloids | 2.83 |
| Trigonelline | Alkaloids | 1.70 |
| Histidinol | Alkaloids | -0.15 |
| DL-2-Aminoadipic acid | Alkaloids | 3.24 |
| Spermine | Phenolamine | -0.15 |
| 1-Methoxy-indole-3-acetamide | Alkaloids | 4.25 |
| Methoxyindoleacetic acid | Plumerane | 4.30 |
| Gallic acid | Tannin | -2.00 |
| Ellagic acid | Tannin | -1.70 |
| Digallic Acid | Tannin | -3.94 |
| 6-O-Galloyl-D-glucose | Tannin | -0.79 |

Continued

| 1-O-Galloyl-β-D-glucose | Tannin | -1.05 |
| --- | --- | --- |
| Galloyl Methyl gallate | Tannin | -4.22 |
| Ellagic acid-4-O-Xyloside | Tannin | 1.51 |
| Gemin D | Tannin | -2.43 |
| Corilagin | Tannin | -1.57 |
| Sanguiin H4 | Tannin | -0.76 |
| Sanguiin H1 | Tannin | -1.32 |
| Tellimagrandin I | Tannin | -0.84 |
| Tercatain | Tannin | -0.98 |
| 1,2,3,6-Tetra-O-Galloyl-D-Glucose | Tannin | -0.64 |
| 1,2,3,4,6-Penta-O-Galloyl-D-Glucose | Tannin | -3.67 |
| L-Valine | Amino acids and derivatives | 1.00 |
| L-Threonine | Amino acids and derivatives | 2.01 |
| Trans-4-Hydroxy-L-proline | Amino acids and derivatives | 2.76 |
| L-Isoleucine | Amino acids and derivatives | 2.46 |
| L-Leucine | Amino acids and derivatives | 2.54 |
| L-Asparagine | Amino acids and derivatives | 3.57 |
| L-Ornithine | Amino acids and derivatives | 3.62 |
| L-Aspartic Acid | Amino acids and derivatives | 1.26 |
| L-Glutamic acid | Amino acids and derivatives | -0.75 |
| L-Tyrosine | Amino acids and derivatives | 1.92 |
| L-Tryptophan | Amino acids and derivatives | 4.25 |
| Glutathione reduced form | Amino acids and derivatives | 8.41 |
| Scopoletin (7-Hydroxy-5-methoxycoumarin) | Coumarins | 3.81 |
| Pinoresinol-4-O-glucoside | Lignans | 2.08 |
| 1-methylguanidine | Nucleotides and derivatives | -1.36 |
| Adenosine | Nucleotides and derivatives | -3.56 |
| Guanosine | Nucleotides and derivatives | -2.53 |
| 5'-Deoxy-5'-(methylthio)adenosine | Nucleotides and derivatives | -2.09 |
| 2'-Deoxyinosine-5'-monophosphate | Nucleotides and derivatives | -1.32 |
| Adenosine 5'-monophosphate | Nucleotides and derivatives | -1.98 |
| Succinyladenosine | Nucleotides and derivatives | 2.69 |
| Uridine 5'-diphospho-D-glucose | Nucleotides and derivatives | 0.62 |
| 3-Hydroxybutyric acid | Organic acids | -0.60 |
| Malonic acid | Organic acids | -0.61 |
| 6-Aminocaproic acid | Organic acids | 2.78 |
| 3-Ureidopropionic Acid | Organic acids | 4.68 |
| L-Malic acid | Organic acids | 0.48 |
| 2,3-Dihydroxy-3-Methylbutanoic Acid | Organic acids | 0.32 |
| 2-Methylglutaric acid | Organic acids | -2.46 |
| Adipic Acid | Organic acids | -2.52 |
| 2,4,6-Trihydroxybenzoic acid | Organic acids | -0.75 |
| Citric Acid | Organic acids | -1.78 |
| Isocitric Acid | Organic acids | -1.48 |
| Quinic Acid | Organic acids | -1.76 |
| Choline Alfoscerate | PC | 3.17 |
| 9,16-Dihydroxypalmitic acid | Free fatty acids | -2.81 |
| L-Ascorbic acid (Vitamin C) | Vitamin | 8.80 |
| D-Glucose | Saccharides and Alcohols | 0.34 |
| D-Pantothenic Acid | Vitamin | 1.33 |
| Methyl Brevifolincarboxylate | Others | -2.76 |
| D-Trehalose | Saccharides and Alcohols | 2.14 |
| Galactinol | Saccharides and Alcohols | -0.78 |

Continued

| Sweroside | Sesquiterpenoids | 4.52 |
| --- | --- | --- |
| 2α-Hydroxyursolic acid | Triterpene | 5.78 |

Table S6. Contents of carotenoids extracted from rose petals at the two stages.

| **Compound** | **Class** | **JT-T** **(μg/g FW)** | **JT-P (μg/g FW)** |
| --- | --- | --- | --- |
| α-carotene | carotenes | 0.77 ± 0.08 | N/A |
| β-carotene | carotenes | 29.96 ± 3.87 | 1.29 ± 0.11 |
| (E/Z)-phytoene | carotenes | 1.40 ± 0.23 | 7.35 ± 0.77 |
| β-cryptoxanthin | xanthophylls | 7.51 ± 0.67 | 0.65 ± 0.01 |
| antheraxanthin | xanthophylls | 0.39 ± 0.04 | 0.11 ± 0.02 |
| 8'-apo-beta-carotenal | xanthophylls | 0.04 ± 0.01 | 0.01 ± 0.001 |
| astaxanthin | xanthophylls | 0.04 ± 0.002 | 0.07 ± 0.01 |
| lutein | xanthophylls | 11.08 ± 1.48 | 7.92 ± 0.45 |
| neoxanthin | xanthophylls | 3.81 ± 0.14 | 0.85 ± 0.03 |
| violaxanthin | xanthophylls | 5.75 ± 0.46 | 2.19 ± 0.17 |
| zeaxanthin | xanthophylls | 9.62 ± 1.19 | 0.92 ± 0.06 |
| canthaxanthin | xanthophylls | 0.002 ± 0.0001 | 0.003 ± 0.0004 |
| echinenone | xanthophylls | 0.011 ± 0.001 | 0.004 ± 0.0008 |
| β-citraurin | xanthophylls | 0.003 ± 2.4E-05 | N/A |
| violaxanthin palmitate | xanthophylls | 38.75 ± 3.45 | 6.40 ± 0.22 |
| violaxanthin-myristate-palmitate | xanthophylls | 10.73 ± 1.50 | 2.83 ± 0.15 |
| violaxanthin-myristate-oleate | xanthophylls | 0.33 ± 0.03 | N/A |
| violaxanthin-myristate-laurate | xanthophylls | 29.83 ± 0.82 | 4.35 ± 0.28 |
| violaxanthin-myristate-caprate | xanthophylls | 44.13 ± 4.86 | N/A |
| violaxanthin myristate | xanthophylls | 51.73 ± 3.83 | 2.74 ± 0.21 |
| violaxanthin laurate | xanthophylls | N/A | 1.03 ± 0.11 |
| violaxanthin dipalmitate | xanthophylls | 2.96 ± 0.20 | 0.64 ± 0.05 |
| violaxanthin dilaurate | xanthophylls | 37.91 ± 4.36 | 9.90 ± 0.08 |
| violaxanthin dimyristate | xanthophylls | 4.02 ± 0.61 | 1.33 ± 0.11 |
| violaxanthin dibutyrate | xanthophylls | 0.02 ± 0.001 | N/A |
| zeaxanthin dimyristate | xanthophylls | 0.24 ± 0.04 | N/A |
| zeaxanthin dipalmitate | xanthophylls | 0.85 ± 0.16 | 0.04 ± 0.003 |
| zeaxanthin-myristate-palmitate | xanthophylls | 0.22 ± 0.02 | N/A |
| zeaxanthin-laurate-palmitate | xanthophylls | 0.26 ± 0.02 | N/A |
| zeaxanthin-palmitate-stearate | xanthophylls | 0.42 ± 0.03 | 0.02 ± 0.002 |
| zeaxanthin dilaurate | xanthophylls | 0.17 ± 0.003 | 0.01 ± 0.001 |

Continued

| zeaxanthin-laurate-myristate | xanthophylls | 0.07 ± 0.02 | N/A |
| --- | --- | --- | --- |
| lutein caprate | xanthophylls | 0.07 ± 0.003 | N/A |
| lutein dilaurate | xanthophylls | 3.48 ± 0.17 | 0.134 ± 0.01 |
| lutein dimyristate | xanthophylls | 1.27 ± 0.02 | 0.07 ± 0.01 |
| lutein dioleate | xanthophylls | 0.43 ± 0.01 | N/A |
| lutein dipalmitate | xanthophylls | 2.20 ± 0.18 | 0.06 ± 0.003 |
| lutein distearate | xanthophylls | 0.75 ± 0.02 | 0.03 ± 0.01 |
| lutein oleate | xanthophylls | 0.09 ± 0.01 | N/A |
| lutein palmitate | xanthophylls | 0.43 ± 0.07 | 0.07 ± 0.01 |
| β-cryptoxanthin laurate | xanthophylls | 2.38 ± 0.41 | 0.09 ± 0.02 |
| β-cryptoxanthin myristate | xanthophylls | 1.03 ± 0.16 | N/A |
| β-cryptoxanthin oleate | xanthophylls | 0.13 ± 0.01 | N/A |
| β-cryptoxanthin palmitate | xanthophylls | 2.64 ± 0.25 | N/A |
| rubixanthin caprate | xanthophylls | 0.35 ± 0.04 | N/A |
| rubixanthin laurate | xanthophylls | 2.65 ± 0.50 | 0.09 ± 0.02 |
| rubixanthin palmitate | xanthophylls | 3.34 ± 0.30 | N/A |
| antheraxanthin dipalmitate | xanthophylls | 1.06 ± 0.12 | N/A |
| neochrome palmitate | xanthophylls | 2.31 ± 0.09 | N/A |

Table S7. The FPKM values of gene expression related to carotenoids metabolic pathway in rose petals.

| **Genes** | **JT-T** | **JT-O** | **JT-P** |
| --- | --- | --- | --- |
| RchiOBHmChr2g0152351 | 613.87 | 113.18 | 28.54 |
| RchiOBHmChr3g0488531 | 34.50 | 39.48 | 20.89 |
| RchiOBHmChr4g0411571 | 155.30 | 135.66 | 67.45 |
| RchiOBHmChr4g0410061 | 86.83 | 43.31 | 14.36 |
| RchiOBHmChr3g0451921 | 447.20 | 386.34 | 109.28 |
| RchiOBHmChr3g0477381 | 31.06 | 16.73 | 9.50 |
| RchiOBHmChr7g0220531 | 1.59 | 4.80 | 17.43 |
| RchiOBHmChr2g0125001 | 20.49 | 17.21 | 17.41 |
| RchiOBHmChr2g0093831 | 1.47 | 9.13 | 18.34 |
| RchiOBHmChr1g0378281 | 18.94 | 10.77 | 24.05 |
| RchiOBHmChr7g0196841 | 192.45 | 103.75 | 52.36 |
| RchiOBHmChr3g0471231 | 19.48 | 9.48 | 16.45 |
| RchiOBHmChr3g0448661 | 6.96 | 40.35 | 49.73 |
| RchiOBHmChr2g0105701 | 46.52 | 59.95 | 39.11 |
| RchiOBHmChr5g0014331 | 43.24 | 6.45 | 1.55 |
| RchiOBHmChr5g0027901 | 3.17 | 1.73 | 5.33 |
| RchiOBHmChr3g0487411 | 3.45 | 20.18 | 14.69 |
| RchiOBHmChr6g0289711 | 0.09 | 1.00 | 0.35 |
| RchiOBHmChr7g0187341 | 0.21 | 8.46 | 8.34 |
| RchiOBHmChr3g0448381 | 41.42 | 9.78 | 17.74 |
| RchiOBHmChr1g0348141 | 1.75 | 27.85 | 5.22 |
| RchiOBHmChr4g0444051 | 8.23 | 550.64 | 772.05 |
| RchiOBHmChr1g0319721 | 1.06 | 1.69 | 4.34 |
| RchiOBHmChr5g0049511 | 3.84 | 8.07 | 13.61 |

Table S8. The FPKM values of gene expression related to anthocyanin biosynthesis pathway in rose petals.

| **Genes** | **JT-T** | **JT-O** | **JT-P** |
| --- | --- | --- | --- |
| RchiOBHmChr1g0363011 | 39.05 | 47.33 | 45.55 |
| RchiOBHmChr3g0469861 | 126.37 | 248.03 | 266.08 |
| RchiOBHmChr7g0212181 | 8.95 | 0.94 | 0.16 |
| RchiOBHmChr5g0073351 | 145.40 | 178.28 | 190.37 |
| RchiOBHmChr1g0384231 | 51.44 | 130.12 | 101.20 |
| RchiOBHmChr2g0131501 | 4.96 | 9.77 | 4.43 |
| RchiOBHmChr4g0402711 | 133.59 | 143.18 | 36.26 |
| RchiOBHmChr1g0316441 | 390.98 | 374.57 | 387.60 |
| RchiOBHmChr1g0316451 | 111.14 | 393.76 | 637.22 |
| RchiOBHmChr1g0316461 | 1061.38 | 1003.27 | 632.98 |
| RchiOBHmChr1g0372181 | 173.13 | 96.08 | 115.97 |
| RchiOBHmChr1g0365111 | 35.84 | 69.41 | 76.12 |
| RchiOBHmChr6g0295121 | 34.43 | 79.58 | 53.56 |
| RchiOBHmChr7g0182961 | 1.55 | 0.38 | 2.70 |
| RchiOBHmChr7g0202001 | 0.49 | 1.38 | 2.92 |
| RchiOBHmChr7g0201771 | 3.50 | 17.29 | 39.78 |
| RchiOBHmChr6g0301421 | 105.26 | 40.02 | 28.92 |
| RchiOBHmChr6g0307561 | 0.61 | 1.72 | 1.29 |
| RchiOBHmChr6g0307711 | 11.75 | 22.63 | 17.02 |
| RchiOBHmChr5g0049051 | 63.72 | 43.51 | 24.37 |
| RchiOBHmChr6g0302721 | 8.57 | 34.43 | 69.72 |
| RchiOBHmChr1g0383951 | 0.33 | 3.90 | 8.21 |
| RchiOBHmChr2g0102291 | 1.73 | 4.58 | 6.07 |
| RchiOBHmChr2g0102311 | 1.14 | 2.16 | 5.44 |
| RchiOBHmChr2g0102591 | 1.95 | 1.97 | 0.29 |
| RchiOBHmChr2g0153231 | 0.27 | 5.07 | 9.67 |
| RchiOBHmChr5g0046041 | 0.60 | 5.55 | 20.34 |
| RchiOBHmChr1g0378941 | 419.81 | 38.79 | 12.38 |
| RchiOBHmChr2g0152971 | 4.75 | 5.64 | 4.79 |
| RchiOBHmChr7g0199941 | 150.98 | 88.63 | 262.22 |
| RchiOBHmChr5g0004611 | 3.42 | 3.32 | 7.72 |
| RchiOBHmChr7g0195261 | 32.76 | 18.26 | 20.33 |
| RchiOBHmChr4g0435881 | 33.85 | 69.08 | 63.07 |
| RchiOBHmChr2g0099421 | 188.02 | 283.36 | 216.34 |

Table S9. The correlation between transcripts and metabolites related to carotenoids in rose petals.

| **Genes ID** | **Symbol** | **Metabolites** | **cor** | **p_value** |
| --- | --- | --- | --- | --- |
| RchiOBHmChr2g0106361 | WRKY7 | Carotenoid_04 | -0.988307156 | 0.000204285 |
| RchiOBHmChr3g0448721 | MYB1 | Carotenoid_04 | 0.988895993 | 0.000184264 |
| RchiOBHmChr3g0457291 | BHLH149 | Carotenoid_04 | -0.985921047 | 0.00029593 |
| RchiOBHmChr4g0445091 | BHLH094 | Carotenoid_04 | 0.988465806 | 0.000198789 |
| RchiOBHmChr5g0077341 | BHLH122 | Carotenoid_04 | -0.987899623 | 0.000218743 |
| RchiOBHmChr6g0252211 | MYB308 | Carotenoid_04 | 0.990816437 | 0.000126119 |
| RchiOBHmChr6g0304811 | MYB123 | Carotenoid_04 | 0.987158187 | 0.000246309 |
| RchiOBHmChr6g0307001 | MYB73 | Carotenoid_04 | 0.984791579 | 0.000345185 |
| RchiOBHmChr7g0202671 | WRKY21 | Carotenoid_04 | -0.985064407 | 0.000332942 |
| RchiOBHmChr7g0210101 | BHLH71 | Carotenoid_04 | 0.991117003 | 0.000118011 |
| RchiOBHmChr7g0241021 | WRKY35 | Carotenoid_04 | -0.978097483 | 0.000714327 |
| RchiOBHmChr2g0093831 | LUT2 | Carotenoid_04 | -0.98481359 | 0.000344189 |
| RchiOBHmChr2g0152351 | PSY | Carotenoid_04 | 0.989648578 | 0.000160173 |
| RchiOBHmChr3g0451921 | ZDS1 | Carotenoid_04 | 0.989518205 | 0.000164226 |
| RchiOBHmChr3g0477381 | CRTISO | Carotenoid_04 | 0.991879594 | 0.0000986 |
| RchiOBHmChr4g0410061 | Z-ISO | Carotenoid_04 | 0.977109958 | 0.000779934 |
| RchiOBHmChr4g0411571 | PDS | Carotenoid_04 | 0.987913671 | 0.000218236 |
| RchiOBHmChr4g0444051 | CCD4 | Carotenoid_04 | -0.98862984 | 0.000193186 |
| RchiOBHmChr5g0014331 | NCED2 | Carotenoid_04 | 0.987284715 | 0.00024149 |
| RchiOBHmChr5g0049511 | AAO3 | Carotenoid_04 | -0.982063382 | 0.000479698 |
| RchiOBHmChr7g0196841 | BETA-OHASE | Carotenoid_04 | 0.98846597 | 0.000198784 |
| RchiOBHmChr2g0167441 | MYB16 | Carotenoid_04 | -0.983148371 | 0.000423573 |
| RchiOBHmChr2g0106671 | MYB73 | Carotenoid_04 | 0.984975027 | 0.000336929 |
| RchiOBHmChr6g0308731 | MYB73 | Carotenoid_04 | 0.99121584 | 0.000115403 |
| RchiOBHmChr2g0116041 | MYB1 | Carotenoid_04 | -0.982270933 | 0.000468693 |
| RchiOBHmChr2g0106361 | WRKY7 | Carotenoid_06 | 0.988627618 | 0.000193261 |
| RchiOBHmChr3g0448721 | MYB1 | Carotenoid_06 | -0.988210023 | 0.000207686 |
| RchiOBHmChr3g0457291 | BHLH149 | Carotenoid_06 | 0.988269937 | 0.000205585 |
| RchiOBHmChr4g0445091 | BHLH094 | Carotenoid_06 | -0.98787226 | 0.000219731 |
| RchiOBHmChr5g0077341 | BHLH122 | Carotenoid_06 | 0.977321789 | 0.00076562 |
| RchiOBHmChr6g0252211 | MYB308 | Carotenoid_06 | -0.984783543 | 0.000345549 |
| RchiOBHmChr6g0304811 | MYB123 | Carotenoid_06 | -0.987725865 | 0.000225057 |

Continued

| RchiOBHmChr6g0307001 | MYB73 | Carotenoid_06 | -0.990215666 | 0.000143131 |
| --- | --- | --- | --- | --- |
| RchiOBHmChr7g0202671 | WRKY21 | Carotenoid_06 | 0.983664176 | 0.000398109 |
| RchiOBHmChr7g0210101 | BHLH71 | Carotenoid_06 | -0.986034832 | 0.000291177 |
| RchiOBHmChr7g0241021 | WRKY35 | Carotenoid_06 | 0.970420081 | 0.001299517 |
| RchiOBHmChr2g0093831 | LUT2 | Carotenoid_06 | 0.990698931 | 0.000129363 |
| RchiOBHmChr2g0152351 | PSY | Carotenoid_06 | -0.987991597 | 0.000215437 |
| RchiOBHmChr3g0451921 | ZDS1 | Carotenoid_06 | -0.987184574 | 0.0002453 |
| RchiOBHmChr3g0477381 | CRTISO | Carotenoid_06 | -0.987604888 | 0.000229506 |
| RchiOBHmChr4g0410061 | Z-ISO | Carotenoid_06 | -0.985518514 | 0.000313052 |
| RchiOBHmChr4g0411571 | PDS | Carotenoid_06 | -0.987457675 | 0.000234978 |
| RchiOBHmChr4g0444051 | CCD4 | Carotenoid_06 | 0.986427751 | 0.000275059 |
| RchiOBHmChr5g0014331 | NCED2 | Carotenoid_06 | -0.990247232 | 0.000142211 |
| RchiOBHmChr5g0049511 | AAO3 | Carotenoid_06 | 0.995370324 | 0.0000321 |
| RchiOBHmChr7g0196841 | BETA-OHASE | Carotenoid_06 | -0.986832469 | 0.000258934 |
| RchiOBHmChr2g0167441 | MYB16 | Carotenoid_06 | 0.981870446 | 0.000490042 |
| RchiOBHmChr2g0106671 | MYB73 | Carotenoid_06 | -0.980611153 | 0.000560247 |
| RchiOBHmChr6g0308731 | MYB73 | Carotenoid_06 | -0.988433906 | 0.000199888 |
| RchiOBHmChr2g0116041 | MYB1 | Carotenoid_06 | 0.978553959 | 0.000684967 |
| RchiOBHmChr2g0106361 | WRKY7 | Carotenoid_60 | -0.993643402 | 0.0000605 |
| RchiOBHmChr3g0448721 | MYB1 | Carotenoid_60 | 0.994186473 | 0.0000506 |
| RchiOBHmChr3g0457291 | BHLH149 | Carotenoid_60 | -0.991686086 | 0.000103394 |
| RchiOBHmChr4g0445091 | BHLH094 | Carotenoid_60 | 0.993871228 | 0.0000562 |
| RchiOBHmChr5g0077341 | BHLH122 | Carotenoid_60 | -0.992420008 | 0.000086 |
| RchiOBHmChr6g0252211 | MYB308 | Carotenoid_60 | 0.99534991 | 0.0000324 |
| RchiOBHmChr6g0304811 | MYB123 | Carotenoid_60 | 0.992919591 | 0.000075 |
| RchiOBHmChr6g0307001 | MYB73 | Carotenoid_60 | 0.99111176 | 0.00011815 |
| RchiOBHmChr7g0202671 | WRKY21 | Carotenoid_60 | -0.990646057 | 0.000130835 |
| RchiOBHmChr7g0210101 | BHLH71 | Carotenoid_60 | 0.99565555 | 0.0000283 |
| RchiOBHmChr7g0241021 | WRKY35 | Carotenoid_60 | -0.983121082 | 0.000424942 |
| RchiOBHmChr2g0093831 | LUT2 | Carotenoid_60 | -0.990035837 | 0.000148432 |
| RchiOBHmChr2g0152351 | PSY | Carotenoid_60 | 0.994733996 | 0.0000415 |
| RchiOBHmChr3g0451921 | ZDS1 | Carotenoid_60 | 0.9945837 | 0.0000439 |
| RchiOBHmChr3g0477381 | CRTISO | Carotenoid_60 | 0.995641227 | 0.0000285 |
| RchiOBHmChr4g0410061 | Z-ISO | Carotenoid_60 | 0.985030437 | 0.000334454 |
| RchiOBHmChr4g0411571 | PDS | Carotenoid_60 | 0.993408736 | 0.000065 |
| RchiOBHmChr4g0444051 | CCD4 | Carotenoid_60 | -0.993774664 | 0.000058 |
| RchiOBHmChr5g0014331 | NCED2 | Carotenoid_60 | 0.992934256 | 0.0000747 |
| RchiOBHmChr5g0049511 | AAO3 | Carotenoid_60 | -0.98746291 | 0.000234783 |
| RchiOBHmChr7g0196841 | BETA-OHASE | Carotenoid_60 | 0.993727614 | 0.0000589 |
| RchiOBHmChr2g0167441 | MYB16 | Carotenoid_60 | -0.989332169 | 0.000170097 |
| RchiOBHmChr2g0106671 | MYB73 | Carotenoid_60 | 0.990524056 | 0.000134265 |

| RchiOBHmChr6g0308731 | MYB73 | Carotenoid_60 | 0.995393495 | 3.1781E-05 |
| --- | --- | --- | --- | --- |
| RchiOBHmChr2g0116041 | MYB1 | Carotenoid_60 | -0.98734776 | 0.000239106 |
| RchiOBHmChr2g0106361 | WRKY7 | Carotenoid_58 | -0.997747069 | 0.00000761 |
| RchiOBHmChr3g0448721 | MYB1 | Carotenoid_58 | 0.998233882 | 0.00000468 |
| RchiOBHmChr3g0457291 | BHLH149 | Carotenoid_58 | -0.997383816 | 0.0000103 |
| RchiOBHmChr4g0445091 | BHLH094 | Carotenoid_58 | 0.998383371 | 0.00000392 |
| RchiOBHmChr5g0077341 | BHLH122 | Carotenoid_58 | -0.99729294 | 0.000011 |
| RchiOBHmChr6g0252211 | MYB308 | Carotenoid_58 | 0.999661932 | 0.000000171 |
| RchiOBHmChr6g0304811 | MYB123 | Carotenoid_58 | 0.998257334 | 0.00000455 |
| RchiOBHmChr6g0307001 | MYB73 | Carotenoid_58 | 0.996759568 | 0.0000157 |
| RchiOBHmChr7g0202671 | WRKY21 | Carotenoid_58 | -0.995262647 | 0.0000336 |
| RchiOBHmChr7g0210101 | BHLH71 | Carotenoid_58 | 0.999639808 | 0.000000195 |
| RchiOBHmChr7g0241021 | WRKY35 | Carotenoid_58 | -0.989087338 | 0.00017798 |
| RchiOBHmChr2g0093831 | LUT2 | Carotenoid_58 | -0.992966704 | 0.000074 |
| RchiOBHmChr2g0152351 | PSY | Carotenoid_58 | 0.998509431 | 0.00000333 |
| RchiOBHmChr3g0451921 | ZDS1 | Carotenoid_58 | 0.999201491 | 0.000000956 |
| RchiOBHmChr3g0477381 | CRTISO | Carotenoid_58 | 0.998621314 | 0.00000285 |
| RchiOBHmChr4g0410061 | Z-ISO | Carotenoid_58 | 0.99426811 | 0.0000492 |
| RchiOBHmChr4g0411571 | PDS | Carotenoid_58 | 0.998823234 | 0.00000208 |
| RchiOBHmChr4g0444051 | CCD4 | Carotenoid_58 | -0.998198651 | 0.00000486 |
| RchiOBHmChr5g0014331 | NCED2 | Carotenoid_58 | 0.996290988 | 0.0000206 |
| RchiOBHmChr5g0049511 | AAO3 | Carotenoid_58 | -0.988131052 | 0.000210472 |
| RchiOBHmChr7g0196841 | BETA-OHASE | Carotenoid_58 | 0.99784218 | 0.00000698 |
| RchiOBHmChr2g0167441 | MYB16 | Carotenoid_58 | -0.996857761 | 1.4795E-05 |
| RchiOBHmChr2g0106671 | MYB73 | Carotenoid_58 | 0.998195333 | 4.8823E-06 |
| RchiOBHmChr6g0308731 | MYB73 | Carotenoid_58 | 0.995672521 | 2.80501E-05 |
| RchiOBHmChr2g0116041 | MYB1 | Carotenoid_58 | -0.992510384 | 8.39315E-05 |
| RchiOBHmChr2g0106361 | WRKY7 | Carotenoid_57 | -0.986548281 | 0.000270206 |
| RchiOBHmChr3g0448721 | MYB1 | Carotenoid_57 | 0.98832148 | 0.000203785 |
| RchiOBHmChr3g0457291 | BHLH149 | Carotenoid_57 | -0.983303286 | 0.000415843 |
| RchiOBHmChr4g0445091 | BHLH094 | Carotenoid_57 | 0.987903938 | 0.000218587 |
| RchiOBHmChr5g0077341 | BHLH122 | Carotenoid_57 | -0.986753107 | 0.000262058 |
| RchiOBHmChr6g0252211 | MYB308 | Carotenoid_57 | 0.991016683 | 0.000120687 |
| RchiOBHmChr6g0304811 | MYB123 | Carotenoid_57 | 0.987090116 | 0.000248922 |
| RchiOBHmChr6g0307001 | MYB73 | Carotenoid_57 | 0.983146747 | 0.000423655 |
| RchiOBHmChr7g0202671 | WRKY21 | Carotenoid_57 | -0.988543985 | 0.000196109 |
| RchiOBHmChr7g0210101 | BHLH71 | Carotenoid_57 | 0.990727109 | 0.000128581 |
| RchiOBHmChr7g0241021 | WRKY35 | Carotenoid_57 | -0.973151859 | 0.001071558 |
| RchiOBHmChr2g0093831 | LUT2 | Carotenoid_57 | -0.979320642 | 0.000637032 |
| RchiOBHmChr2g0152351 | PSY | Carotenoid_57 | 0.989238524 | 0.000173091 |
| RchiOBHmChr3g0451921 | ZDS1 | Carotenoid_57 | 0.989004497 | 0.000180687 |
| RchiOBHmChr3g0477381 | CRTISO | Carotenoid_57 | 0.99002861 | 0.000148647 |
| RchiOBHmChr4g0410061 | Z-ISO | Carotenoid_57 | 0.978134961 | 0.000711893 |

Continued

| RchiOBHmChr4g0411571 | PDS | Carotenoid_57 | 0.987536907 | 0.000232025 |
| --- | --- | --- | --- | --- |
| RchiOBHmChr4g0444051 | CCD4 | Carotenoid_57 | -0.986979427 | 0.000253199 |
| RchiOBHmChr5g0014331 | NCED2 | Carotenoid_57 | 0.986763694 | 0.00026164 |
| RchiOBHmChr5g0049511 | AAO3 | Carotenoid_57 | -0.982508721 | 0.000456242 |
| RchiOBHmChr7g0196841 | BETA-OHASE | Carotenoid_57 | 0.987430339 | 0.000236002 |
| RchiOBHmChr2g0167441 | MYB16 | Carotenoid_57 | -0.98172855 | 0.000497719 |
| RchiOBHmChr2g0106671 | MYB73 | Carotenoid_57 | 0.984431166 | 0.000361696 |
| RchiOBHmChr6g0308731 | MYB73 | Carotenoid_57 | 0.991169742 | 0.000116616 |
| RchiOBHmChr2g0116041 | MYB1 | Carotenoid_57 | -0.977405712 | 0.000759986 |
| RchiOBHmChr2g0106361 | WRKY7 | Carotenoid_56 | -0.987068943 | 0.000249737 |
| RchiOBHmChr3g0448721 | MYB1 | Carotenoid_56 | 0.987534372 | 0.000232119 |
| RchiOBHmChr3g0457291 | BHLH149 | Carotenoid_56 | -0.987948635 | 0.000216978 |
| RchiOBHmChr4g0445091 | BHLH094 | Carotenoid_56 | 0.987875082 | 0.000219629 |
| RchiOBHmChr5g0077341 | BHLH122 | Carotenoid_56 | -0.987776611 | 0.000223204 |
| RchiOBHmChr6g0252211 | MYB308 | Carotenoid_56 | 0.992541905 | 0.0000832 |
| RchiOBHmChr6g0304811 | MYB123 | Carotenoid_56 | 0.987669541 | 0.000227123 |
| RchiOBHmChr6g0307001 | MYB73 | Carotenoid_56 | 0.98500565 | 0.00033556 |
| RchiOBHmChr7g0202671 | WRKY21 | Carotenoid_56 | -0.984307592 | 0.000367445 |
| RchiOBHmChr7g0210101 | BHLH71 | Carotenoid_56 | 0.992169111 | 0.0000917 |
| RchiOBHmChr7g0241021 | WRKY35 | Carotenoid_56 | -0.978337955 | 0.000698784 |
| RchiOBHmChr2g0093831 | LUT2 | Carotenoid_56 | -0.983014484 | 0.000430311 |
| RchiOBHmChr2g0152351 | PSY | Carotenoid_56 | 0.988290038 | 0.000204882 |
| RchiOBHmChr3g0451921 | ZDS1 | Carotenoid_56 | 0.99064157 | 0.000130961 |
| RchiOBHmChr3g0477381 | CRTISO | Carotenoid_56 | 0.995456457 | 0.0000309 |
| RchiOBHmChr4g0410061 | Z-ISO | Carotenoid_56 | 0.980368126 | 0.000574333 |
| RchiOBHmChr4g0411571 | PDS | Carotenoid_56 | 0.989864316 | 0.000153578 |
| RchiOBHmChr4g0444051 | CCD4 | Carotenoid_56 | -0.988151138 | 0.000209762 |
| RchiOBHmChr5g0014331 | NCED2 | Carotenoid_56 | 0.983478725 | 0.000407174 |
| RchiOBHmChr5g0049511 | AAO3 | Carotenoid_56 | -0.978705707 | 0.000675342 |
| RchiOBHmChr7g0196841 | BETA-OHASE | Carotenoid_56 | 0.986685578 | 0.000264731 |
| RchiOBHmChr2g0167441 | MYB16 | Carotenoid_56 | -0.985969305 | 0.00029391 |
| RchiOBHmChr2g0106671 | MYB73 | Carotenoid_56 | 0.991224052 | 0.000115188 |
| RchiOBHmChr6g0308731 | MYB73 | Carotenoid_56 | 0.984196943 | 0.000372632 |
| RchiOBHmChr2g0116041 | MYB1 | Carotenoid_56 | -0.982378182 | 0.000463057 |

Note: Carotenoid_04, β-carotene; Carotenoid_06, (E/Z)-phytoene; Carotenoid_56, Zeaxanthin; Carotenoid_57, Violaxanthin; Carotenoid_58, Neoxanthin; Carotenoid_60, β-cryptoxanthin.

Table S10. The correlation between carotenoid-related genes and TF-related genes in rose petals.

| **TF-related genes** | **Carotenoid-related genes** | **cor** | **p_value** |
| --- | --- | --- | --- |
| RchiOBHmChr3g0457291 | RchiOBHmChr2g0093831 | 0.96515237 | 9.80168E-11 |
| RchiOBHmChr4g0445091 | RchiOBHmChr2g0093831 | -0.913539517 | 1.19382E-07 |
| RchiOBHmChr5g0077341 | RchiOBHmChr2g0093831 | 0.951747082 | 1.26963E-09 |
| RchiOBHmChr6g0252211 | RchiOBHmChr2g0093831 | -0.941122909 | 6.0309E-09 |
| RchiOBHmChr7g0202671 | RchiOBHmChr2g0093831 | 0.925394695 | 3.81171E-08 |
| RchiOBHmChr7g0210101 | RchiOBHmChr2g0093831 | -0.904840451 | 2.49926E-07 |
| RchiOBHmChr7g0241021 | RchiOBHmChr2g0093831 | 0.900507715 | 3.51893E-07 |
| RchiOBHmChr2g0106361 | RchiOBHmChr2g0152351 | -0.868817786 | 2.89841E-06 |
| RchiOBHmChr2g0167441 | RchiOBHmChr2g0152351 | 0.800375277 | 6.63311E-05 |
| RchiOBHmChr3g0457291 | RchiOBHmChr2g0152351 | -0.870062342 | 2.6966E-06 |
| RchiOBHmChr4g0445091 | RchiOBHmChr2g0152351 | 0.834060384 | 1.69345E-05 |
| RchiOBHmChr5g0077341 | RchiOBHmChr2g0152351 | -0.899120055 | 3.91362E-07 |
| RchiOBHmChr6g0252211 | RchiOBHmChr2g0152351 | 0.859287189 | 4.92262E-06 |
| RchiOBHmChr6g0304811 | RchiOBHmChr2g0152351 | 0.877658154 | 1.70748E-06 |
| RchiOBHmChr6g0307001 | RchiOBHmChr2g0152351 | 0.94819393 | 2.21632E-09 |
| RchiOBHmChr7g0210101 | RchiOBHmChr2g0152351 | 0.89613032 | 4.89592E-07 |
| RchiOBHmChr7g0241021 | RchiOBHmChr2g0152351 | -0.952387021 | 1.14333E-09 |
| RchiOBHmChr2g0106361 | RchiOBHmChr3g0451921 | -0.889872844 | 7.66017E-07 |
| RchiOBHmChr3g0448721 | RchiOBHmChr3g0451921 | 0.993413665 | 1.74413E-16 |
| RchiOBHmChr6g0252211 | RchiOBHmChr3g0451921 | 0.863985996 | 3.81E-06 |
| RchiOBHmChr2g0106671 | RchiOBHmChr3g0477381 | -0.882435848 | 1.26108E-06 |
| RchiOBHmChr2g0167441 | RchiOBHmChr3g0477381 | 0.909792273 | 1.65614E-07 |
| RchiOBHmChr3g0448721 | RchiOBHmChr3g0477381 | 0.884954018 | 1.06927E-06 |
| RchiOBHmChr6g0252211 | RchiOBHmChr3g0477381 | 0.813020088 | 4.10113E-05 |
| RchiOBHmChr6g0304811 | RchiOBHmChr3g0477381 | 0.907698021 | 1.97649E-07 |
| RchiOBHmChr6g0307001 | RchiOBHmChr3g0477381 | 0.912259485 | 1.33723E-07 |
| RchiOBHmChr2g0106361 | RchiOBHmChr4g0410061 | -0.919789959 | 6.68301E-08 |
| RchiOBHmChr2g0167441 | RchiOBHmChr4g0410061 | 0.836158632 | 1.54028E-05 |
| RchiOBHmChr3g0448721 | RchiOBHmChr4g0410061 | 0.935520815 | 1.22575E-08 |
| RchiOBHmChr6g0252211 | RchiOBHmChr4g0410061 | 0.895794414 | 5.01855E-07 |
| RchiOBHmChr6g0304811 | RchiOBHmChr4g0410061 | 0.865877895 | 3.42739E-06 |
| RchiOBHmChr6g0307001 | RchiOBHmChr4g0410061 | 0.929957473 | 2.33459E-08 |
| RchiOBHmChr7g0241021 | RchiOBHmChr4g0410061 | -0.815568273 | 3.70641E-05 |
| RchiOBHmChr2g0106361 | RchiOBHmChr4g0411571 | -0.954118563 | 8.54774E-10 |
| RchiOBHmChr3g0448721 | RchiOBHmChr4g0411571 | 0.916987395 | 8.7172E-08 |
| RchiOBHmChr3g0457291 | RchiOBHmChr4g0411571 | -0.864720172 | 3.65738E-06 |
| RchiOBHmChr4g0445091 | RchiOBHmChr4g0411571 | 0.912393286 | 1.32158E-07 |
| RchiOBHmChr6g0252211 | RchiOBHmChr4g0411571 | 0.855915077 | 5.88354E-06 |
| RchiOBHmChr7g0210101 | RchiOBHmChr4g0411571 | 0.822284162 | 2.81756E-05 |
| RchiOBHmChr2g0106671 | RchiOBHmChr4g0444051 | 0.969664172 | 3.27899E-11 |
| RchiOBHmChr2g0116041 | RchiOBHmChr4g0444051 | 0.820402987 | 3.0459E-05 |

Continued

| RchiOBHmChr6g0308731 | RchiOBHmChr4g0444051 | 0.857440138 | 5.43071E-06 |
| --- | --- | --- | --- |
| RchiOBHmChr7g0210101 | RchiOBHmChr5g0014331 | 0.881621589 | 1.32913E-06 |
| RchiOBHmChr2g0106361 | RchiOBHmChr5g0049511 | 0.922146899 | 5.30445E-08 |
| RchiOBHmChr3g0457291 | RchiOBHmChr5g0049511 | 0.829088002 | 2.1093E-05 |
| RchiOBHmChr4g0445091 | RchiOBHmChr5g0049511 | -0.938393938 | 8.59049E-09 |
| RchiOBHmChr6g0252211 | RchiOBHmChr5g0049511 | -0.865558507 | 3.48956E-06 |
| RchiOBHmChr7g0210101 | RchiOBHmChr5g0049511 | -0.850891821 | 7.61173E-06 |
| RchiOBHmChr2g0106361 | RchiOBHmChr7g0196841 | -0.813482088 | 4.02702E-05 |
| RchiOBHmChr2g0106671 | RchiOBHmChr7g0196841 | -0.897725901 | 4.3481E-07 |
| RchiOBHmChr3g0448721 | RchiOBHmChr7g0196841 | 0.897214771 | 4.51751E-07 |
| RchiOBHmChr6g0252211 | RchiOBHmChr7g0196841 | 0.843626716 | 1.08719E-05 |

Table S11. The correlation between transcripts and metabolites related to flavonoids in rose petals.

| **Genes ID** | **Symbol** | **Metabolites** | **cor** | **p_value** |
| --- | --- | --- | --- | --- |
| RchiOBHmChr1g0378941 | RhGT1 | mws1094 | 0.992965615 | 7.40E-05 |
| RchiOBHmChr1g0316451 | PKS1 | mws1094 | -0.992869597 | 7.61E-05 |
| RchiOBHmChr1g0383951 | FGT | mws1094 | -0.988137522 | 0.000210243 |
| RchiOBHmChr1g0365111 | CHI2 | mws1094 | -0.988075017 | 0.00021246 |
| RchiOBHmChr1g0383951 | FGT | mws0091 | 0.900947996 | 0.014231035 |
| RchiOBHmChr1g0378941 | RhGT1 | mws0091 | -0.883550911 | 0.019551038 |
| RchiOBHmChr1g0316451 | PKS1 | mws0091 | 0.880038692 | 0.020722908 |
| RchiOBHmChr1g0365111 | CHI2 | mws0091 | 0.87008207 | 0.024221582 |
| RchiOBHmChr1g0383951 | FGT | pme1793 | -0.834267307 | 0.038924871 |
| RchiOBHmChr1g0378941 | RhGT1 | pme1793 | 0.829856086 | 0.040960683 |
| RchiOBHmChr1g0316451 | PKS1 | pme1793 | -0.828570722 | 0.041563015 |
| RchiOBHmChr2g0167441 | MYB16 | mws1094 | 0.996333078 | 2.01E-05 |
| RchiOBHmChr2g0106671 | MYB73 | mws1094 | -0.990849299 | 0.00012522 |
| RchiOBHmChr2g0153231 | UGT71K1 | mws1094 | -0.989441127 | 0.000166646 |
| RchiOBHmChr2g0106671 | MYB73 | mws0091 | 0.902238487 | 0.013868801 |
| RchiOBHmChr2g0167441 | MYB16 | mws0091 | -0.885143911 | 0.019030296 |
| RchiOBHmChr2g0153231 | UGT71K1 | mws0091 | 0.882513516 | 0.019893774 |
| RchiOBHmChr2g0167441 | MYB16 | pme1793 | 0.858231595 | 0.02872277 |
| RchiOBHmChr2g0106671 | MYB73 | pme1793 | -0.847181542 | 0.0332458 |
| RchiOBHmChr2g0153231 | UGT71K1 | pme1793 | -0.809526162 | 0.050965202 |
| RchiOBHmChr3g0457291 | BHLH149 | mws1094 | -0.994563228 | 4.43E-05 |
| RchiOBHmChr6g0307001 | MYB73 | mws1094 | 0.994375505 | 4.74E-05 |
| RchiOBHmChr4g0402711 | 4CL2 | mws1094 | 0.994135564 | 5.15E-05 |
| RchiOBHmChr6g0302721 | F3GT2 | mws1094 | -0.99326856 | 6.78E-05 |
| RchiOBHmChr6g0252211 | MYB308 | mws1094 | 0.990661288 | 0.00013041 |
| RchiOBHmChr5g0049051 | GT5 | mws1094 | 0.992585682 | 8.23E-05 |
| RchiOBHmChr4g0445091 | BHLH094 | mws1094 | 0.991862665 | 9.91E-05 |
| RchiOBHmChr3g0448721 | MYB1 | mws1094 | 0.991057219 | 0.000119602 |
| RchiOBHmChr6g0301421 | DFR | mws1094 | 0.99073352 | 0.000128404 |
| RchiOBHmChr3g0469861 | PAL1 | mws1094 | -0.990705552 | 0.000129179 |
| RchiOBHmChr5g0046041 | RhGT1 | mws1094 | -0.990561958 | 0.000133195 |
| RchiOBHmChr6g0308731 | MYB73 | mws1094 | -0.988738942 | 0.000189503 |
| RchiOBHmChr5g0004611 | ANR | mws1094 | -0.981841291 | 0.000491614 |
| RchiOBHmChr5g0077341 | BHLH122 | mws1094 | -0.988255193 | 0.000206101 |
| RchiOBHmChr4g0441081 | MYB4 | mws1094 | -0.986375426 | 0.000277179 |
| RchiOBHmChr7g0228621 | MYB305 | mws1094 | -0.983891572 | 0.000387132 |
| RchiOBHmChr4g0441081 | MYB4 | mws0091 | 0.881162424 | 0.02034442 |
| RchiOBHmChr7g0228621 | MYB305 | mws0091 | 0.855736611 | 0.029716689 |
| RchiOBHmChr4g0441081 | MYB4 | pme1793 | -0.849471043 | 0.032283035 |
| RchiOBHmChr7g0228621 | MYB305 | pme1793 | -0.887292203 | 0.018338705 |

Continued

| RchiOBHmChr5g0077341 | BHLH122 | mws0091 | 0.90083232 | 0.014263724 |
| --- | --- | --- | --- | --- |
| RchiOBHmChr5g0004611 | ANR | mws0091 | 0.911529683 | 0.011394267 |
| RchiOBHmChr5g0049051 | GT5 | mws0091 | -0.901248055 | 0.014146408 |
| RchiOBHmChr3g0469861 | PAL1 | mws0091 | 0.888443059 | 0.017973266 |
| RchiOBHmChr4g0402711 | 4CL2 | mws0091 | -0.887934123 | 0.018134437 |
| RchiOBHmChr6g0252211 | MYB308 | mws0091 | -0.900807892 | 0.014270632 |
| RchiOBHmChr4g0445091 | BHLH094 | mws0091 | -0.886103243 | 0.018719946 |
| RchiOBHmChr3g0448721 | MYB1 | mws0091 | -0.885228144 | 0.019002948 |
| RchiOBHmChr6g0301421 | DFR | mws0091 | -0.881010162 | 0.020395509 |
| RchiOBHmChr3g0457291 | BHLH149 | mws0091 | 0.879557902 | 0.020885864 |
| RchiOBHmChr6g0302721 | F3GT2 | mws0091 | 0.877480709 | 0.021596898 |
| RchiOBHmChr6g0308731 | MYB73 | mws0091 | 0.874641111 | 0.022587278 |
| RchiOBHmChr6g0307001 | MYB73 | mws0091 | -0.873404073 | 0.023025346 |
| RchiOBHmChr5g0046041 | RhGT1 | mws0091 | 0.873297038 | 0.023063439 |
| RchiOBHmChr3g0457291 | BHLH149 | pme1793 | -0.850109007 | 0.032017141 |
| RchiOBHmChr6g0302721 | F3GT2 | pme1793 | -0.841358161 | 0.035754561 |
| RchiOBHmChr6g0307001 | MYB73 | pme1793 | 0.839235367 | 0.036690398 |
| RchiOBHmChr5g0049051 | GT5 | pme1793 | 0.839115375 | 0.036743636 |
| RchiOBHmChr4g0402711 | 4CL2 | pme1793 | 0.837922155 | 0.037275012 |
| RchiOBHmChr5g0046041 | RhGT1 | pme1793 | -0.83790172 | 0.037284143 |
| RchiOBHmChr6g0252211 | MYB308 | pme1793 | 0.828954604 | 0.041382694 |
| RchiOBHmChr5g0077341 | BHLH122 | pme1793 | -0.845783562 | 0.033840222 |
| RchiOBHmChr4g0445091 | BHLH094 | pme1793 | 0.829967292 | 0.040908764 |
| RchiOBHmChr3g0469861 | PAL1 | pme1793 | -0.827764798 | 0.041942772 |
| RchiOBHmChr6g0301421 | DFR | pme1793 | 0.827014254 | 0.042297884 |
| RchiOBHmChr3g0448721 | MYB1 | pme1793 | 0.826622496 | 0.042483796 |
| RchiOBHmChr5g0004611 | ANR | pme1793 | -0.816905407 | 0.047216447 |
| RchiOBHmChr7g0210101 | BHLH71 | mws1094 | 0.990819344 | 0.00012604 |
| RchiOBHmChr7g0201771 | CYP75B137 | mws1094 | -0.987287616 | 0.00024138 |
| RchiOBHmChr7g0212181 | PAL1 | mws1094 | 0.987161142 | 0.000246196 |
| RchiOBHmChr2g0116041 | MYB1 | mws1094 | -0.988173807 | 0.000208961 |
| RchiOBHmChr7g0201771 | CYP75B137 | mws0091 | 0.899331334 | 0.014691173 |
| RchiOBHmChr7g0210101 | BHLH71 | mws0091 | -0.897313365 | 0.015275426 |
| RchiOBHmChr7g0212181 | PAL1 | mws0091 | -0.885226991 | 0.019003322 |
| RchiOBHmChr2g0116041 | MYB1 | mws0091 | 0.878427064 | 0.021271546 |
| RchiOBHmChr7g0210101 | BHLH71 | pme1793 | 0.829969349 | 0.040907804 |
| RchiOBHmChr7g0212181 | PAL1 | pme1793 | 0.813245212 | 0.04905927 |
| RchiOBHmChr7g0201771 | CYP75B137 | pme1793 | -0.811856655 | 0.049766936 |
| RchiOBHmChr2g0116041 | MYB1 | pme1793 | -0.864878713 | 0.026153137 |

Note: pme1793, pelargonidin 3, 5-diglucoside (Pg3G5G); mws1094, dihydrokaempfero; mws0091, isoquercetin.

Table S12. The correlation between flavonoid-related genes and TF-related genes in the rose petals.

| **TF-related genes** | **Flavonoid-related genes** | **cor** | **p_value** |
| --- | --- | --- | --- |
| RchiOBHmChr2g0167441 | RchiOBHmChr1g0378941 | 0.934305344 | 1.42E-08 |
| RchiOBHmChr6g0252211 | RchiOBHmChr1g0378941 | 0.809829324 | 4.65E-05 |
| RchiOBHmChr6g0304811 | RchiOBHmChr1g0378941 | 0.967015067 | 6.35E-11 |
| RchiOBHmChr6g0307001 | RchiOBHmChr1g0378941 | 0.961842599 | 2.00E-10 |
| RchiOBHmChr2g0116041 | RchiOBHmChr1g0383951 | 0.959825599 | 3.01E-10 |
| RchiOBHmChr6g0308731 | RchiOBHmChr1g0383951 | 0.859367407 | 4.90E-06 |
| RchiOBHmChr7g0228621 | RchiOBHmChr1g0383951 | 0.987464985 | 2.95E-14 |
| RchiOBHmChr2g0106671 | RchiOBHmChr2g0153231 | 0.890352376 | 7.41E-07 |
| RchiOBHmChr2g0116041 | RchiOBHmChr3g0469861 | 0.903517182 | 2.78E-07 |
| RchiOBHmChr4g0441081 | RchiOBHmChr3g0469861 | 0.843858507 | 1.08E-05 |
| RchiOBHmChr6g0308731 | RchiOBHmChr3g0469861 | 0.845095249 | 1.01E-05 |
| RchiOBHmChr7g0228621 | RchiOBHmChr3g0469861 | 0.883580872 | 1.17E-06 |
| RchiOBHmChr3g0448721 | RchiOBHmChr4g0402711 | 0.953953231 | 8.79E-10 |
| RchiOBHmChr3g0457291 | RchiOBHmChr5g0046041 | 0.826551137 | 2.35E-05 |
| RchiOBHmChr2g0167441 | RchiOBHmChr5g0049051 | 0.811474671 | 4.36E-05 |
| RchiOBHmChr3g0448721 | RchiOBHmChr5g0049051 | 0.963598357 | 1.38E-10 |
| RchiOBHmChr6g0252211 | RchiOBHmChr5g0049051 | 0.849623605 | 8.11E-06 |
| RchiOBHmChr6g0304811 | RchiOBHmChr5g0049051 | 0.815707622 | 3.69E-05 |
| RchiOBHmChr6g0307001 | RchiOBHmChr5g0049051 | 0.862523727 | 4.13E-06 |
| RchiOBHmChr2g0116041 | RchiOBHmChr6g0301421 | 0.926970706 | 3.23E-08 |
| RchiOBHmChr6g0308731 | RchiOBHmChr6g0301421 | 0.826169477 | 2.39E-05 |
| RchiOBHmChr7g0228621 | RchiOBHmChr6g0301421 | 0.966640604 | 6.95E-11 |
| RchiOBHmChr3g0457291 | RchiOBHmChr6g0302721 | 0.909670596 | 1.67E-07 |
| RchiOBHmChr5g0077341 | RchiOBHmChr6g0302721 | 0.909447456 | 1.71E-07 |
| RchiOBHmChr3g0457291 | RchiOBHmChr7g0201771 | 0.892107974 | 6.55E-07 |
| RchiOBHmChr5g0077341 | RchiOBHmChr7g0201771 | 0.847858779 | 8.85E-06 |
| RchiOBHmChr4g0445091 | RchiOBHmChr7g0212181 | 0.819350734 | 3.18E-05 |
| RchiOBHmChr6g0252211 | RchiOBHmChr7g0212181 | 0.845706101 | 9.84E-06 |
| RchiOBHmChr6g0304811 | RchiOBHmChr7g0212181 | 0.881448453 | 1.34E-06 |
| RchiOBHmChr6g0307001 | RchiOBHmChr7g0212181 | 0.949877469 | 1.71E-09 |
| RchiOBHmChr7g0210101 | RchiOBHmChr7g0212181 | 0.887702007 | 8.89E-07 |
| RchiOBHmChr2g0167441 | RchiOBHmChr1g0316451 | -0.894460185 | 5.53219E-07 |
| RchiOBHmChr6g0304811 | RchiOBHmChr1g0316451 | -0.809278456 | 4.74533E-05 |
| RchiOBHmChr6g0252211 | RchiOBHmChr1g0365111 | -0.875902341 | 1.90269E-06 |
| RchiOBHmChr2g0106671 | RchiOBHmChr4g0402711 | -0.802621259 | 6.10529E-05 |
| RchiOBHmChr2g0172331 | RchiOBHmChr5g0046041 | -0.912117745 | 1.35399E-07 |
| RchiOBHmChr4g0445091 | RchiOBHmChr5g0046041 | -0.842051682 | 1.17179E-05 |
| RchiOBHmChr6g0252211 | RchiOBHmChr5g0046041 | -0.902333617 | 3.05229E-07 |
| RchiOBHmChr2g0106671 | RchiOBHmChr5g0049051 | -0.894707047 | 5.43387E-07 |
| RchiOBHmChr4g0445091 | RchiOBHmChr6g0302721 | -0.852356189 | 7.06805E-06 |
| RchiOBHmChr7g0210101 | RchiOBHmChr6g0302721 | -0.912924438 | 1.26098E-07 |
| RchiOBHmChr2g0172331 | RchiOBHmChr7g0201771 | -0.901482337 | 3.26272E-07 |

Continued

| RchiOBHmChr4g0445091 | RchiOBHmChr7g0201771 | -0.890210574 | 7.48246E-07 |
| --- | --- | --- | --- |
| RchiOBHmChr6g0252211 | RchiOBHmChr7g0201771 | -0.934750253 | 1.34464E-08 |
| RchiOBHmChr7g0210101 | RchiOBHmChr7g0201771 | -0.831383288 | 1.90763E-05 |
| RchiOBHmChr3g0457291 | RchiOBHmChr7g0212181 | -0.851266563 | 7.4693E-06 |
| RchiOBHmChr5g0077341 | RchiOBHmChr7g0212181 | -0.884436728 | 1.10647E-06 |

Table S13. Diferentially expressed transcription factors (TF) among JT-T, JT-O and JT-P of the roses.

| Gene | JT-T_mean  (FPKM Value) | JT-O_mean (FPKM Value) | JT-P_mean (FPKM Value) |
| --- | --- | --- | --- |
| RchiOBHmChr2g0116041 | 0.54 | 32.49 | 114.60 |
| RchiOBHmChr3g0448721 | 730.36 | 551.71 | 64.06 |
| RchiOBHmChr2g0167441 | 55.81 | 19.21 | 15.36 |
| RchiOBHmChr2g0172331 | 390.49 | 347.33 | 76.29 |
| RchiOBHmChr7g0228621 | 37.94 | 98.38 | 76.31 |
| RchiOBHmChr4g0441081 | 68.90 | 192.95 | 144.23 |
| RchiOBHmChr2g0106671 | 22.11 | 54.21 | 70.80 |
| RchiOBHmChr6g0307001 | 42.06 | 5.53 | 3.30 |
| RchiOBHmChr6g0308731 | 72.94 | 221.34 | 157.05 |
| RchiOBHmChr6g0304811 | 197.24 | 3.74 | 4.57 |
| RchiOBHmChr7g0210101 | 147.78 | 57.62 | 9.76 |
| RchiOBHmChr3g0457291 | 9.95 | 33.86 | 66.72 |
| RchiOBHmChr5g0077341 | 4.23 | 20.55 | 33.96 |
| RchiOBHmChr4g0445091 | 330.98 | 214.50 | 56.16 |
| RchiOBHmChr2g0106361 | 4.18 | 26.33 | 42.51 |
| RchiOBHmChr7g0202671 | 0.20 | 19.09 | 62.14 |
| RchiOBHmChr7g0241021 | 6.54 | 61.85 | 88.87 |
| RchiOBHmChr6g0252211 | 98.83 | 54.76 | 14.67 |

Table S14. TFs related to pigment metabolism in roses best hit with Arabidopsis.

| Gene | Symbol | Blast Best Hit |  |
| --- | --- | --- | --- |
|  |  | AGI | Description |
| RchiOBHmChr2g0116041 | MYB1 | AT1G66370 | ATMYB113; Involved in regulation of anthocyanin biosynthesis. |
| RchiOBHmChr3g0448721 | MYB1 | AT1G66380.1 | AtMYB114; Involved in regulation of anthocyanin biosynthesis. |
| RchiOBHmChr2g0167441 | MYB16 | AT3G01140.2 | myb domain protein 106; Encodes a MIXTA-like MYB gene NOECK. |
| RchiOBHmChr2g0172331 | MYB305 | AT3G27810.1 | Encodes a member of the R2R3-MYB. Promotes flavonol biosynthesis. |
| RchiOBHmChr7g0228621 | MYB330 | AT4G09460.1 | AtMYB6; myb domain protein 6. |
| RchiOBHmChr4g0441081 | MYB4 | AT4G38620.1 | AtMYB4; a suppressor of the cinnamate 4-hydroxylase (C4H) gene |
| RchiOBHmChr2g0106671 | MYB73 | AT4G37260.1 | myb domain protein 73; Member of the R2R3 factor gene family. |
| RchiOBHmChr6g0307001 | MYB73 | AT4G37260.1 | myb domain protein 73; Member of the R2R3 factor gene family. |
| RchiOBHmChr6g0308731 | MYB73 | AT4G37260.1 | myb domain protein 73; Member of the R2R3 factor gene family. |
| RchiOBHmChr6g0304811 | MYB123 | AT1G22640.1 | myb domain protein 3. |
| RchiOBHmChr6g0252211 | MYB308 | AT4G38620.1 | AtMYB4; a suppressor of the cinnamate 4-hydroxylase (C4H) gene |
| RchiOBHmChr7g0210101 | BHLH71 | AT1G72210.1 | basic helix-loop-helix (bHLH) DNA-binding superfamily protein |
| RchiOBHmChr3g0457291 | BHLH149 | AT1G09250.1 | ATBS1 Interacting Factor 4  basic helix-loop-helix (bHLH) DNA-binding superfamily protein |
| RchiOBHmChr5g0077341 | BHLH122 | AT1G51140.1 | AtCFL1 associated protein 1, FLOWERING BHLH 3, ABA-responsive kinase substrate |
| RchiOBHmChr4g0445091 | BHLH094 | AT1G59640.1 | BIG Petal UB, BIG PETAL P, BIG PETAL |
| RchiOBHmChr2g0106361 | WRKY7 | AT4G24240 | WRKY DNA-binding protein 7  Encodes a Ca-dependent calmodulin binding protein |
| RchiOBHmChr7g0202671 | WRKY21 | AT2G30590 | Encodes WRKY DNA-binding protein 21 |
| RchiOBHmChr7g0241021 | WRKY35 | AT2G34830.1 | maternal effect embryo arrest 24, WRKY DNA-binding protein 35 |
